# The impact of sustained malaria control in the Loreto region of Peru: a retrospective, observational, spatially-varying interrupted time series analysis of the PAMAFRO program

**DOI:** 10.1016/j.lana.2023.100477

**Published:** 2023-03-16

**Authors:** Mark M. Janko, G. Cristina Recalde-Coronel, Camila P. Damasceno, Gabriela Salmón-Mulanovich, Alisson F. Barbieri, Andrés G. Lescano, Benjamin F. Zaitchik, William K. Pan

**Affiliations:** a Duke Global Health Institute, Duke University, Durham, NC, USA; b Institute for Health Metrics and Evaluation, University of Washington, Seattle, WA, USA; c Department of Earth and Planetary Sciences, Johns Hopkins University, Baltimore, MD, USA; d Facultad de Ingeniería Marítima y Ciencias del Mar, Escuela Superior Politécnica del Litoral, Guayaquil, Ecuador; e Universidade Federal de Minas Gerais (UFMG), Belo Horizonte, Brazil; f Pontifica Universidad Catolica del Peru, Lima, Peru; g Clima, Latin American Center of Excellence for Climate Change and Health, and Emerge, Emerging Diseases and Climate Change Research Unit, Universidad Peruana Cayetano Heredia, Lima, Peru; h Nicholas School of the Environment, Duke University, Durham, NC, USA

**Keywords:** Malaria, PAMAFRO, Climate, Interrupted time series, Spatially-varying coefficients, Loreto

## Abstract

**Background:**

Although malaria control investments worldwide have resulted in dramatic declines in transmission since 2000, progress has stalled. In the Amazon, malaria resurgence has followed withdrawal of Global Fund support of the Project for Malaria Control in Andean Border Areas (PAMAFRO). We estimate intervention-specific and spatially-explicit effects of the PAMAFRO program on malaria incidence across the Loreto region of Peru, and consider the influence of the environmental risk factors in the presence of interventions.

**Methods:**

We conducted a retrospective, observational, spatial interrupted time series analysis of malaria incidence rates among people reporting to health posts across Loreto, Peru between the first epidemiological week of January 2001 and the last epidemiological week of December 2016. Model inference is at the smallest administrative unit (district), where the weekly number of diagnosed cases of *Plasmodium vivax* and *Plasmodium falciparum* were determined by microscopy. Census data provided population at risk. We include as covariates weekly estimates of minimum temperature and cumulative precipitation in each district, as well as spatially- and temporally-lagged malaria incidence rates. Environmental data were derived from a hydrometeorological model designed for the Amazon. We used Bayesian spatiotemporal modeling techniques to estimate the impact of the PAMAFRO program, variability in environmental effects, and the role of climate anomalies on transmission after PAMAFRO withdrawal.

**Findings:**

During the PAMAFRO program, incidence of *P. vivax* declined from 42.8 to 10.1 cases/1000 people/year. Incidence for *P. falciparum* declined from 14.3 to 2.5 cases/1000 people/year over this same period. The effects of PAMAFRO-supported interventions varied both by geography and species of malaria. Interventions were only effective in districts where interventions were also deployed in surrounding districts. Further, interventions diminished the effects of other prevailing demographic and environmental risk factors. Withdrawal of the program led to a resurgence in transmission. Increasing minimum temperatures and variability and intensity of rainfall events from 2011 onward and accompanying population displacements contributed to this resurgence.

**Interpretation:**

Malaria control programs must consider the climate and environmental scope of interventions to maximize effectiveness. They must also ensure financial sustainability to maintain local progress and commitment to malaria prevention and elimination efforts, as well as to offset the effects of environmental change that increase transmission risk.

**Funding:**

10.13039/100000104National Aeronautics and Space Administration, 10.13039/100000002National Institutes of Health, 10.13039/100000865Bill and Melinda Gates Foundation.

**Research in context**

**Evidence before this study**

Progress in reducing malaria transmission worldwide has stalled, with some areas of the world experiencing resurgences following long-term reductions in transmission. Loreto, Peru is one such region, with malaria resurgence beginning directly after the end of Global Fund support for its Project for Malaria Control in Andean Border Areas (PAMAFRO). A previous evaluation of the program found that it was associated with significant declines in transmission but did not estimate effects of the different interventions that made up the program, nor did it address the environmental drivers of seasonal transmission or how intervention effects might vary geographically due to variability in implementation or differences in background ecology. As a result, the evidence produced in prior work cannot inform the local control strategies that are needed to successfully control and eliminate malaria.

**Added value of this study**

This study improves on the prior assessment of the PAMAFRO program's impact on malaria incidence between 2006 and 2010 by assessing intervention-specific effects, accounting for seasonal variability in transmission, geographic variability in intervention implementation, and the corresponding geographic variability in intervention effects. Because the average temperature in Peru has increased by 0.5C in the past 20 years, this study further assesses the impact of climate on malaria, particularly the resurgence following the end of the PAMAFRO program. We consider whether interventions were able to buffer environmental effects on transmission.

**Implications of all the available evidence**

Our results show that sustained malaria control led to dramatic reductions in malaria incidence across the region overall, but intervention effects varied both geographically and by *Plasmodium* species. Interventions exhibited the greatest effectiveness in districts that were surrounded by districts that also received intervention support. Further, results demonstrate that reductions cannot be maintained in the absence of ongoing support. External programmatic funding for malaria control must include an evaluation of the implementation environment prior to withdrawal to avert possible future malaria resurgence.

## Introduction

Global efforts to reduce malaria resulted in the total annual incidence falling from an estimated 81 cases per 1000 population at risk in 2000 to 59 in 2020.^1^ Central to the success of this reduction has been a financial commitment from international donors to scale up interventions such as long-lasting insecticidal nets (LLINs), indoor residual spraying (IRS), improved diagnostics using rapid diagnostic tests (RDTs), and treatment with artemisinin combination therapies.^2^ For example, between 2000 and 2011, international funding for malaria control grew from 100 million USD to 1.84 billion USD.^3^^,^^4^ Since 2011, however, international funding levels have risen slowly to 3.0 billion USD in 2019, far short of the 5.6 billion USD the WHO estimates is needed to eliminate the disease.^5^ The WHO has credited this lack of funding commitment for variable transmission across regions, together with increasing climate and environmental variability, among other factors.^5^

In the Americas, almost 90% of malaria is reported in the Amazon, where interventions reduced reported cases from 1.6 million in 2000 to 615,177 in 2011.^1^ However, since 2011, malaria began to rebound and reductions achieved over the prior decade vanished, with the largest increases observed in Brazil, Colombia, Ecuador, Peru, and Venezuela.^6^ Several factors likely contributed to this increase, including a strong El Nino Southern Oscillation (ENSO) in 2011–12 that produced favorable conditions for transmission, such as displacement of over 70,000 people due to flooding, deterioration of health infrastructure, and increased breeding sites for *An. darlingi* mosquitos, the dominant vector in the region.^7^ That same year also marked the end of the Project for Malaria Control in Andean Border Areas (PAMAFRO), which was supported by the Global Fund and provided primary funding for comprehensive malaria control in Venezuela, Colombia, Ecuador, and Peru from 2006-2010.^7^ In 2005, the year prior to the program, 345,062 malaria cases were reported across PAMAFRO regions, declining to a historical low of 175,837 by 2011.^1^ However, by 2015, malaria cases returned to pre-intervention levels (361,428) and the accomplishments of PAMAFRO were no longer observable.^1^

Following the end of the PAMAFRO program in Peru, the Ministry of Health was unable to maintain the same level of intervention support due to lack of human and financial resources.^6^ PAMAFRO's end corresponded with the Global Fund shifting its funding priorities to focus on countries with the highest disease burden and the fewest resources, resulting in steep declines in overall malaria spending among middle-income countries with a lower malaria burden compared to most of sub-Saharan Africa.^3^ Thus, the rebound in the Amazon may have been caused by decreased funding levels due to de-prioritization of malaria burden in the Amazon by international donors and the failure of local-global partnerships in effectively implementing a viable transition plan. Should this be the case, it would have consequences for the future implementation of malaria control and elimination strategies worldwide.^5^^,^^8^ It not only highlights the need to ensure that malaria control measures are deployed where they are most effective, but that local-global partnerships must evaluate the implementation environment to more effectively transfer ownership of programs. Thus, it is critical to understand localized estimates of the PAMAFRO intervention.

The PAMAFRO program supported four intervention areas: 1) distributing LLINs and insecticide re-treatment kits; 2) strengthening malaria diagnostics through distribution of RDTs and new microscopes, while also maintaining existing microscopes and training microscopists; 3) improving malaria case management through the distribution of antimalarial drugs and training of health workers; and 4) promoting community environmental management.^9^ Soto et al. conducted an analysis of PAMAFRO in between 2002 and 2013, demonstrating success of the program and, following funding withdrawal, rapid resurgence of malaria to pre-intervention levels. Although novel, results from Soto et al. have important limitations in terms of guiding improvements to malaria control. First, the authors assume intervention effects were constant over space. This assumption introduces bias in estimating the intervention attributable risk, particularly because the four interventions were implemented heterogeneously over space and time. Further, the authors do not provide estimates of intervention effectiveness for each district they were deployed, limiting the ability of decision-makers to take context into account when deciding where future intervention efforts should be targeted. Second, the authors also assumed that interventions were only effective during the years they were deployed. However, every intervention can have time-lagged effects. For example, LLINs are durable for two to three years, and health worker trainings, community engaged environmental management, and diagnostic strengthening all produce local knowledge that can influence transmission trajectories. This simplifying assumption introduces additional bias in estimating intervention effects. Finally, estimates of PAMAFRO's impact relied on aggregate annual malaria incidence rates and annual averages of environmental covariates, meaning the authors could not account for the seasonality of malaria transmission.

The aim of this paper is to quantify the effects of the PAMAFRO program on malaria incidence in the Loreto region of Peru, an ecologically diverse region of roughly one million people that is representative of the broader Amazon and where 90% of Peru's malaria cases are reported. Our study is novel in that we provide district-level estimates for the effects of each of the four PAMAFRO interventions. We further quantify the role of environmental (precipitation, temperature) and demographic factors (human movement) contributing to the spatial and temporal variability of malaria.

## Methods

### Study design and population

We conducted a retrospective, observational study using malaria surveillance data reported weekly across 49 districts in the Loreto region of Peru between January 2001 and December 2016. The primary outcome is the number of *Plasmodium vivax* (*Pv*) and *Plasmodium falciparum* (*Pf*) cases reported in each district during each epidemiological week. We work at the district level because public health decision-making (including PAMAFRO interventions) occurs at the district level. Cases were reported to the *Dirección General de Epidemiología* as part of its disease surveillance activities, which are publicly available (http://www.dge.gob.pe/salasituacional/sala/index/6_mapaCanal/87).^9^ We defined the population at risk in each district using estimates from the Instituto Nacional de Estatística e Informática (INEI). We chose the time period 2001–2016 because it includes the five-year PAMAFRO intervention period, as well as the five-year periods prior to and after the program.

### Intervention data

We obtained intervention data from Soto et al., which recorded the district and year in which each intervention was deployed and ended.^7^ This produced a set of four indicator variables that took the value 1 if the intervention was deployed in a district during a given year, and 0 otherwise. To allow for each intervention to be scaled up and take effect at a population level, we lag the interventions by one year. Further, we considered each intervention to remain in effect one year after it was fully deployed, consistent with previous research.^10^^,^^11^ As a sensitivity analysis, we allow the interventions to take effect the year they were implemented and remain in effect only during the year of deployment, with the exception of LLIN deployments, which are assumed to remain effective for an additional year.

### Included confounders

We included measures of cumulative rainfall (defined as total rainfall in a district 4–8 weeks prior), and weekly minimum temperature (in Celsius). We choose this period of rainfall exposure to allow for the malaria transmission life cycle to be completed, including the development and emergence of adult mosquitos from aquatic habitats and subsequent biting behaviors. We chose minimum temperature because it varied across the range at which larval and adult mosquito populations are either most abundant (22–24 °C) or experience population collapse (e.g. ≤16 °C), and because the maximum and average temperatures were always in the suitable ranges for *Anopheles* and parasite populations. Our use of minimum temperature is also consistent with prior work in the area.^12^^,^^13^ The environmental covariates were produced at the same spatial and temporal resolution as the malaria surveillance data by a Land Data Assimilation System (LDAS) designed and validated for the Amazon region.^14^, ^15^, ^16^, ^17^, ^18^ We also include an indicator for the urban Iquitos district, which we treat as a confounder since urban centers have a different malaria ecology, serve as transportation hubs, and because the regional health directorate (DIRESA) responsible for coordinating interventions is based in the city. Finally, to account for human mobility and its role in malaria transmission, we constructed a spatially- and temporally-lagged exposure defined as the cumulative *P. vivax* or *P. falciparum* incidence in each district's neighboring districts during the prior four weeks.

### Statistical analysis

Because the PAMAFRO program was a population-level intervention with a clearly-defined point in time when and where interventions began and ended, we adopt an interrupted time series (ITS) approach to estimate its impact.^19^ We extend the ITS model to allow intervention and confounder effects to vary spatially using Conditional Autoregressive (CAR) prior distributions.^20^ We further allow confounders to vary temporally using penalized splines.^21^, ^22^, ^23^ We do this to account for the fact that we do not observe the *Anopheles* mosquito population, whose composition and behavior vary across the region and over time, and because previous work has observed temporally varying effects of rainfall and temperature.^13^^,^^24^^,^^25^ Spatially-varying intervention effects also allow interventions in one district to influence neighboring districts, akin to a spillover effect.

We stratified models by malaria species, used a Poisson likelihood, and compared models with the two different intervention coding schemes using WAIC.^26^ All continuous covariates were centered and scaled as follows: cumulative rainfall was scaled such that coefficients correspond to a 15-mm increase in rainfall; minimum temperature was only mean centered, while spatio-temporally lagged incidence rates were scaled such that regression coefficients correspond to an increase of 1 case per 1000 people at risk during the prior month. All models were fit in R using Integrated Nested Laplace Approximation.^27^ Further details are included in the Supplementary Material.

### Ethics approval

This study analyzes publicly available, de-identified, secondary data, and was deemed not to constitute human subjects research by the Duke University IRB.

### Role of funding source

Study funders had no role in the design, data collection, analysis, interpretation, or writing of the report. The authors had full access to all the data in the study and had final responsibility for the decision to publish. We followed STROBE guidelines, which we include in the Supplementary Material.

## Results

**Fig. 1:**
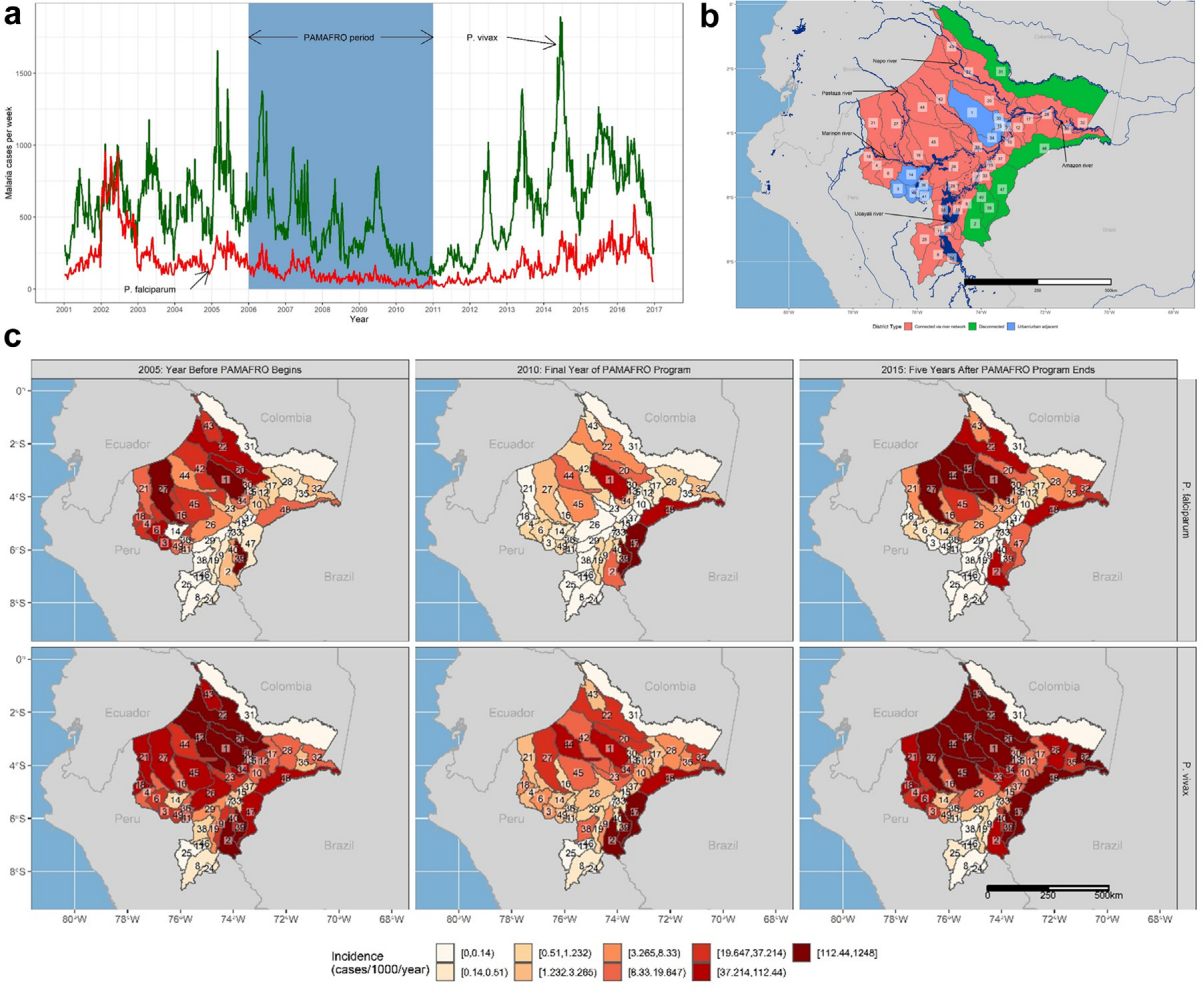
**Malaria transmission across the Loreto region of Peru, 2001–2016**. **(a)** Total *P. vivax* and P. falciparum cases per week from 2001 to 2017 across Loreto. The shaded region corresponds to the 5-year PAMAFRO period. **(b)** Map of the Loreto region. Colors correspond to whether or not the district is an urban center or adjacent to an urban center, connected to urban centers via the river systyem, or disconnected from both urban centers and the river system. Numbers correspond to district names, as follows: 1) Alto Nanay, 2) Alto Tapiche, 3) Balsapuerto, 4) Barranca, 5) Belen, 6) Cahuapanas, 7) Capelo, 8) Contamana, 9) Emilio San Martin, 10) Fernando Lores, 11) Inahuaya, 12) Indiana, 13) Iquitos, 14) Jeberos, 15) Jenaro Herrera, 16) Lagunas, 17) Las Amazonas, 18) Manseriche, 19) Maquia, 20) Mazan, 21) Morona, 22) Napo, 23) Nauta, 24) Padre Marquez, 25) Pampa Hermosa, 26) Parinari, 27) Pastaza, 28) Pebas, 29) Puinahua, 30) Punchana, 31) Putumayo, 32) Ramon Castilla, 33) Requena, 34) San Juan Bautista, 35) San Pablo, 36) Santa Cruz, 37) Saquena, 38) Sarayacu, 39) Soplin, 40) Tapiche, 41) Teniente Cesar Lopez Rojas, 42) Tigre, 43) Torres Causana, 44) Trompeteros, 45) Urarinas, 46) Vargas Guerra, 47) Yaquerana, 48) Yavari, 49) Yurimaguas. **(c)** Annual incidence rates for *P. falciparum* (top) and *P. vivax* (bottom) the year prior to the PAMAFRO program, the final year of the PAMAFRO program, and five years after the program's end.

Malaria cases declined 78% during the PAMAFRO program, from a total of 50,953 cases (38,204 *P. vivax* and 12,749 *P. falciparum*) the year prior to the program to 11,204 cases (8995 *P. vivax*, 2209 *P. falciparum*) during its final year (Fig. 1a), consistent with findings from a prior analysis.^7^ However, our results build on that prior analysis by identifying effects that varied by intervention, species, and district. We further tested assumptions about whether or not interventions were only effective during the years in which they were deployed, and also account for the effects of environmental confounders on intervention effectiveness and transmission.

### Heterogeneity of intervention deployments

**Fig. 2:**
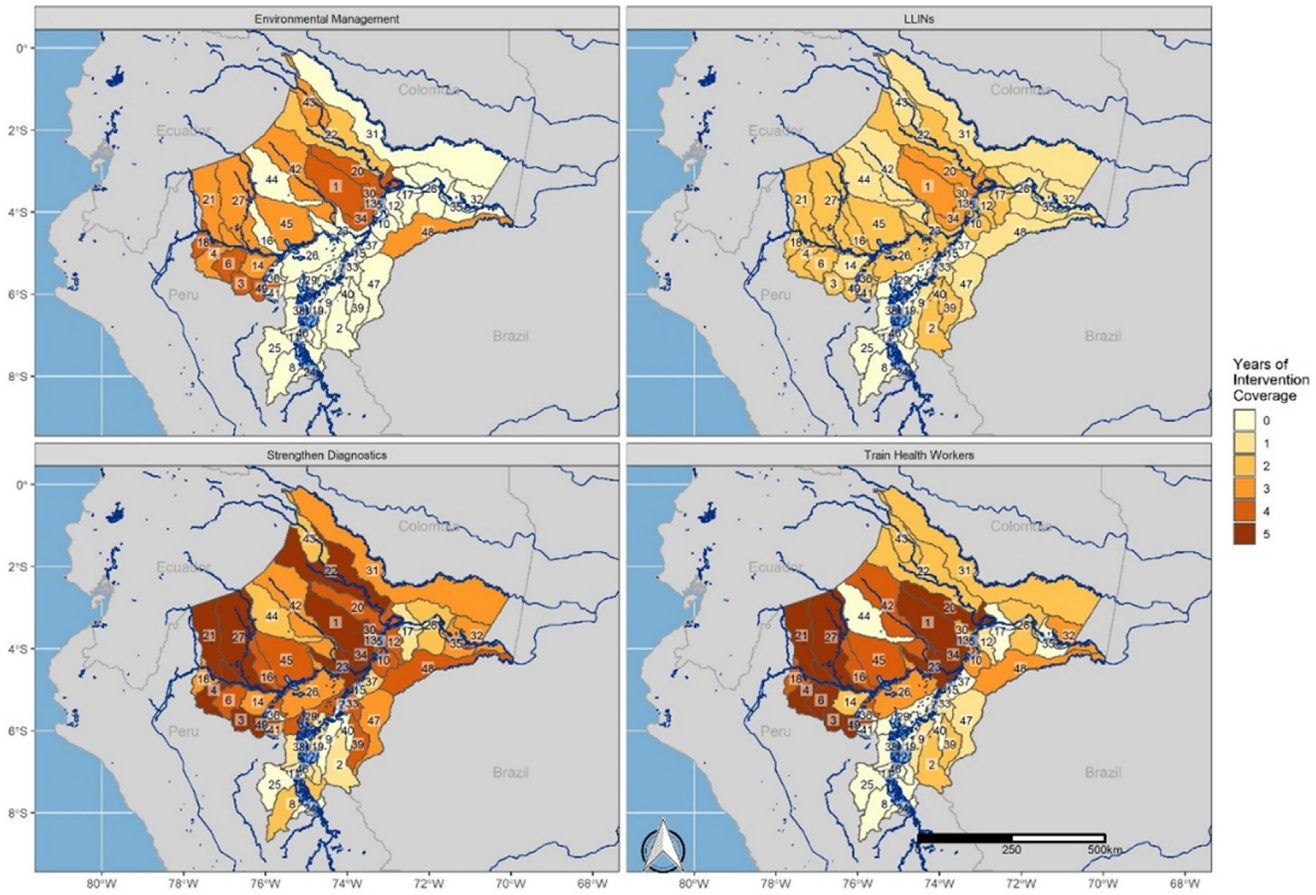
**Intervention coverage by district.** Total years of intervention coverage by district and by intervention. Numbers correspond to district names, as follows: 1) Alto Nanay, 2) Alto Tapiche, 3) Balsapuerto, 4) Barranca, 5) Belen, 6) Cahuapanas, 7) Capelo, 8) Contamana, 9) Emilio San Martin, 10) Fernando Lores, 11) Inahuaya, 12) Indiana, 13) Iquitos, 14) Jeberos, 15) Jenaro Herrera, 16) Lagunas, 17) Las Amazonas, 18) Manseriche, 19) Maquia, 20) Mazan, 21) Morona, 22) Napo, 23) Nauta, 24) Padre Marquez, 25) Pampa Hermosa, 26) Parinari, 27) Pastaza, 28) Pebas, 29) Puinahua, 30) Punchana, 31) Putumayo, 32) Ramon Castilla, 33) Requena, 34) San Juan Bautista, 35) San Pablo, 36) Santa Cruz, 37) Saquena, 38) Sarayacu, 39) Soplin, 40) Tapiche, 41) Teniente Cesar Lopez Rojas, 42) Tigre, 43) Torres Causana, 44) Trompeteros, 45) Urarinas, 46) Vargas Guerra, 47) Yaquerana, 48) Yavari, 49) Yurimaguas.

The four interventions making up the PAMAFRO program were deployed heterogeneously across Loreto, varying by malaria cases, by proximity to the urban districts of Iquitos and Yurimaguas, and by connectivity to the Amazon River and its tributaries (Fig. 2; Supplemental Figs. S1–S4). Overall, 43% of total malaria cases reported during the PAMAFRO program came from the ten districts that included urban centers or were adjacent to them. These districts received 33% of total LLIN deployments, 43% of environmental management deployments, 27% of deployments to strengthen diagnostics, and 30% of deployments to train health workers. Conversely, districts connected to the Amazon and its tributaries reported a total of 47% of malaria cases during PAMAFRO. These districts were targeted by 49% of LLIN deployments, 48% of environmental management deployments, 57% of deployments to strengthen diagnostics, and 55% of deployments to train health workers. Finally, 10% of total cases were reported in districts that were neither connected via the river system nor adjacent to urban centers. Collectively, they were targeted with 18% of LLIN deployments, 9% of environmental management deployments, 16% of deployments to strengthen diagnostics, and 15% of deployments to train health workers. It should be noted, however, that a district receiving a certain level of deployments does not necessarily indicate level of coverage, as our measure of a deployment is a binary indicator for whether or not an intervention was targeted in a given district in a given year.

### Heterogeneity of intervention effectiveness

**Fig. 3:**
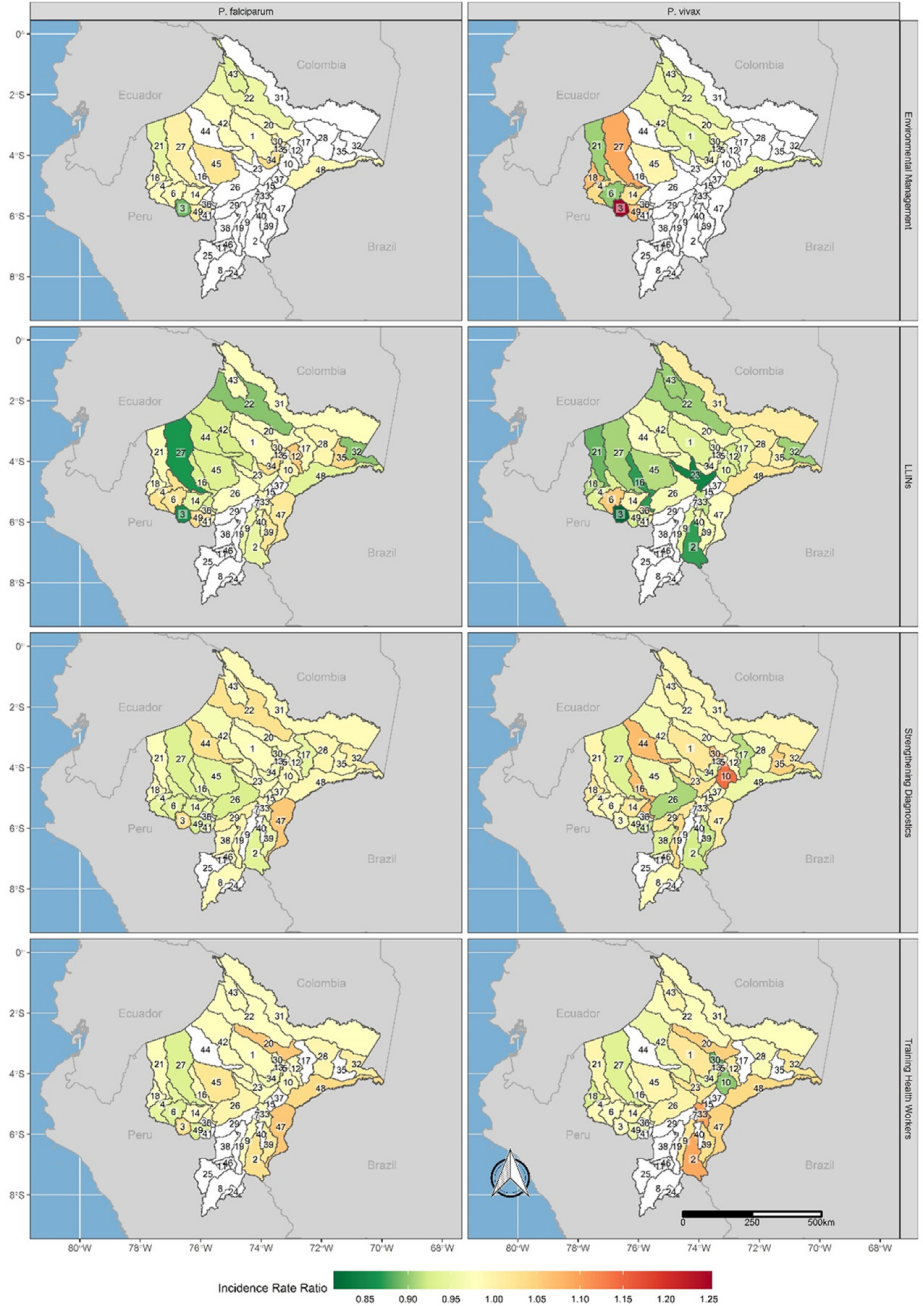
**Incidence rate ratios and uncertainty intervals for each intervention, species, and district.** Annualized Incidence Rate Ratios (IRR) for each intervention and malaria species in each district. Non-shaded districts are those where interventions were not deployed.

The effects of individual interventions varied by district and malaria species (Fig. 3, Supplemental Tables S2–S5). Our model comparison indicated that the effects of the interventions were not immediate but were lagged until fully deployed and remained in effect for at least one year past their initial deployment (Supplemental Table S1). Across all interventions, effectiveness was greatest in districts where the intervention was deployed while also being deployed in adjacent districts. For example, LLIN deployments had the greatest effect in reducing annual incidence of both *P. falciparum* (IRR 0.86, 95% UI 0.84–0.88) and *P. vivax* (IRR 0.81, 95% UI 0.80–0.83) in Balsapuerto, a district in western Loreto that received LLIN deployments during two years of the PAMAFRO program. The three adjacent districts were targeted with a total of five LLIN deployments (two in Cahuapanas, one in Jeberos, and two in Yurimaguas). LLINs were also effective across large swaths of northern Loreto and along the border with Ecuador, as well as areas along the Brazil border. These districts were also adjacent to districts that also received LLIN deployments. We observed similar results for other interventions. For environmental management, the greatest reductions for *P. falciparum* (IRR 0.89, 95% UI 0.88–0.90) and *P. vivax* (IRR 0.90, 95% UI 0.88–0.92) were observed in Balsapuerto and neighboring Cahuapanas, respectively. Additional effects were observed in districts spanning the Napo river corridor and in the far northwest along the Ecuador border. Deployments to strengthen diagnostics were associated with the greatest declines in *P. falciparum* in Yurimaguas (IRR 0.92, 95% UI 0.90–0.95), while for *P. vivax* the greatest effectiveness was observed in Las Amazonas and Teniente Cesar Lopez Rojas (IRR 0.91, 95% UI 0.90–0.93 for both districts). Finally, training health workers was associated with the greatest declines in *P. falciparum* (IRR 0.92, 95% UI 0.90–0.95) in Yurimaguas and in *P. vivax* (IRR 0.88, 95% UI 0.88–0.89) in Punchana, which is adjacent to the capital of Iquitos. In all cases, these districts both received intervention support, and were surrounded by districts that also received support. Further, we did not observe any association with greater levels of surrounding intervention support and intervention effects.

There was only one exception to this: in Yavari, in southeastern Loreto along the border with Brazil, three years of environmental management deployments were associated with 5% annual declines in *P. vivax* incidence rates (IRR 0.95, 95% UI 0.92–0.98). No neighboring district in Loreto received intervention support. However, malaria control has been active since 2005 in the Brazilian municipality of Atalaia do Norte, which borders the Yavari district. Most of the population in this area lives along the border with Peru, while sparsely and low-density indigenous communities inhabit most of the territory, suggesting that interventions in Brazil would have a spill-over effect in neighboring communities in Peru. These interventions may partially explain the decrease in transmission.^28^

Widespread intervention coverage was not a guarantee that a district receiving an intervention while adjacent districts also received the intervention would experience declines in transmission. In a number of settings, null associations or increases in risk were observed. For example, training health workers was associated with increased *P. falciparum* or *P. vivax* incidence in a number of districts, with the greatest increases observed in Mazan (*P. falciparum*; IRR 1.06, 95% UI 1.05–1.07), which is near the capital of Iquitos and surrounded by other districts that received health worker trainings. Similarly, in Alto Tapiche, an isolated district along the Brazil border adjacent to some districts that received interventions, training health workers was associated with significant increases in *P. vivax* (IRR 1.09, 95% UI 1.07–1.11) and *P. falciparum* (IRR 1.03, 95% UI 1.00–1.06). Interventions to strengthen diagnostics were associated with the greatest increases in Yaquerana (*P. falciparum*; IRR 1.06, 95% UI 1.02–1.10), an isolated district along the Brazil border, and Fernando Lores (*P. vivax*; IRR 1.14, 95% UI 1.13–1.16), which straddles the Amazon River near Iquitos. LLIN distributions were associated with the greatest increases in Indiana (*P. falciparum*; IRR 1.06, 95% UI 1.03–1.08), which is connected to Iquitos via the Amazon river, and Cahuapanas (*P. vivax*; IRR 1.05, 95% UI 1.03–1.08), which is connected to the urban district of Yurimaguas via the Mariñon river. Both regions had among the highest levels of LLIN distributions during the study period. Finally, environmental management interventions were associated with the greatest increases in Iquitos (*P. falciparum*; IRR 1.04, 95% UI 1.00–1.07) and Balsapuerto (*P. vivax*; IRR 1.25, 95% UI 1.23–1.28), which is adjacent to the urban district of Yurimaguas in western Loreto. These districts were also adjacent to districts also receiving environmental management support. Finally, we did not observe significant spillover effects, whereby a district receiving no intervention would still experience significant reductions in transmission owing to the interventions being deployed in surrounding districts.

### Interventions as mitigation against climate change

**Fig. 4:**
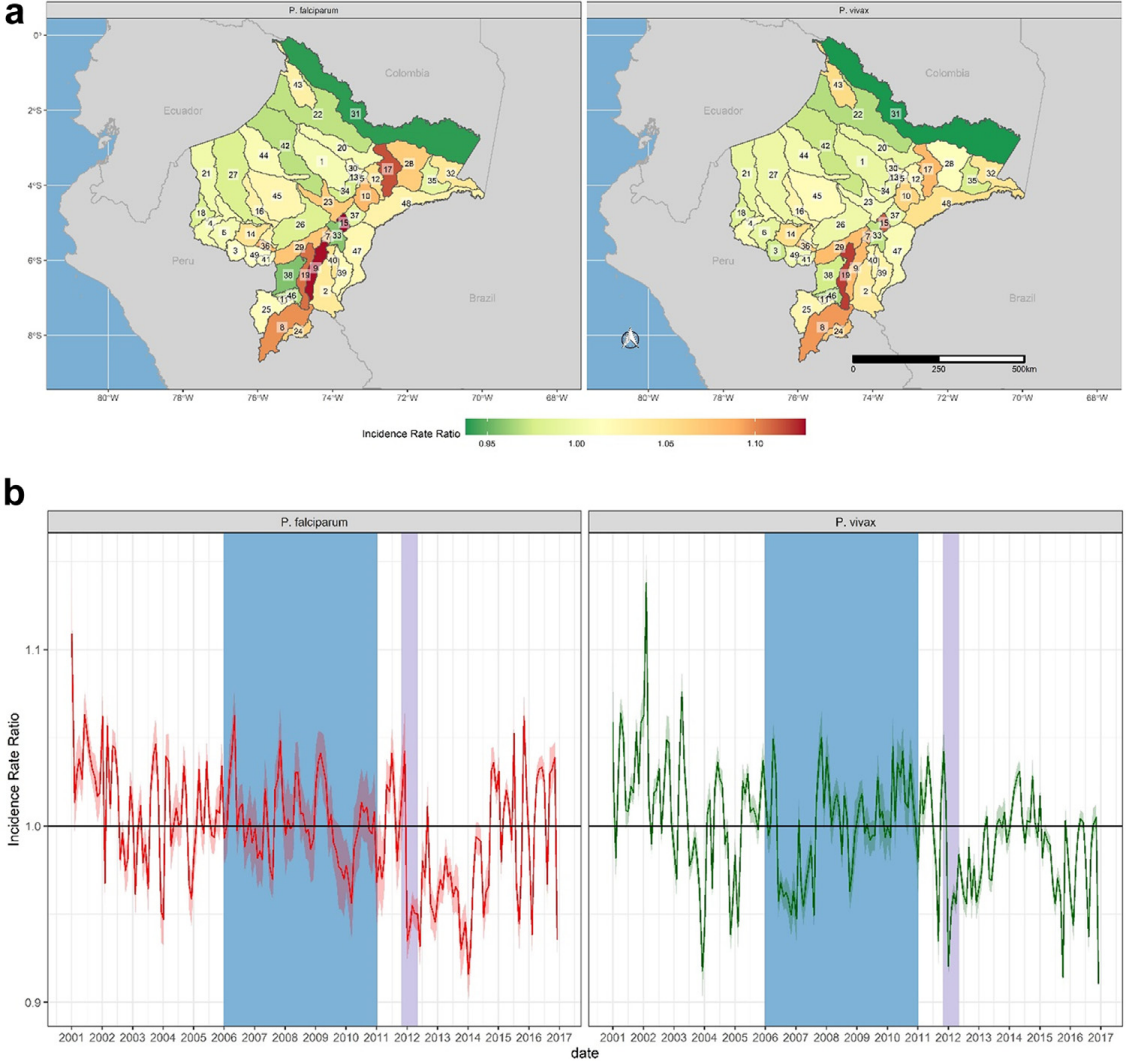
**Spatial and Temporal effects of cumulative rainfall on malaria incidence rates across Loreto.** District-level **(a)** and monthly **(b)** effects of cumulative rainfall on *P. falciparum* (left) and *P. vivax* (right). Incidence Rate Ratios are based on a 15-mm increase in cumulative rainfall between 4 and 8 weeks prior to a given time point. District labels are as follows: 1) Alto Nanay, 2) Alto Tapiche, 3) Balsapuerto, 4) Barranca, 5) Belen, 6) Cahuapanas, 7) Capelo, 8) Contamana, 9) Emilio San Martin, 10) Fernando Lores, 11) Inahuaya, 12) Indiana, 13) Iquitos, 14) Jeberos, 15) Jenaro Herrera, 16) Lagunas, 17) Las Amazonas, 18) Manseriche, 19) Maquia, 20) Mazan, 21) Morona, 22) Napo, 23) Nauta, 24) Padre Marquez, 25) Pampa Hermosa, 26) Parinari, 27) Pastaza, 28) Pebas, 29) Puinahua, 30) Punchana, 31) Putumayo, 32) Ramon Castilla, 33) Requena, 34) San Juan Bautista, 35) San Pablo, 36) Santa Cruz, 37) Saquena, 38) Sarayacu, 39) Soplin, 40) Tapiche, 41) Teniente Cesar Lopez Rojas, 42) Tigre, 43) Torres Causana, 44) Trompeteros, 45) Urarinas, 46) Vargas Guerra, 47) Yaquerana, 48) Yavari, 49) Yurimaguas. The shaded blue region corresponds to the period of the PAMAFRO program. The shaded purple region corresponds to the period of the 2011-12 Loreto floods.

The PAMAFRO program was implemented against a backdrop of environmental variability and change. For example, across Loreto, average cumulative rainfall during the last five years of the study period was 31% higher than during the first five years, while also becoming increasingly variable (Supplemental Fig. S5a). These increases were not uniform across Loreto, leading to spatial variability in the effects of rainfall on transmission (Fig. 4a and Supplemental Figs. S5b and S6). The greatest effects were observed along the Amazon and Ucayali rivers, with the highest incidence rate ratios being observed in Jenaro Herrera (IRR 1.13, 95% UI 1.11–1.15) and Maquia (IRR 1.12, 95% UI 1.10–1.13) districts for *P. falciparum* and *P. vivax*, respectively. The effect of rainfall on transmission also varied over time across Loreto, and for much of the post-PAMAFRO period, cumulative rainfall is associated with lower incidence rate ratios for both *P. vivax* and *P. falciparum* (Fig. 4b), possibly owing to the effects of increasingly extreme weather events on population displacement and the destruction of health infrastructure. This is consistent with findings showing increased effects of spatio-temporally lagged incidence subsequent to the PAMAFRO program. For example, severe flooding from November 2011–April 2012 (Supplemental Figs. S6 and S7) affected over 229,000 residents in Loreto (23% of the population), many of whom were temporarily displaced. Our results indicate a significant increase in incidence due to spatio-temporally lagged incidence towards the end of the flood period (Fig. 6b), suggesting that displacement due to severe flooding contributed to transmission.^29^

**Fig. 5:**
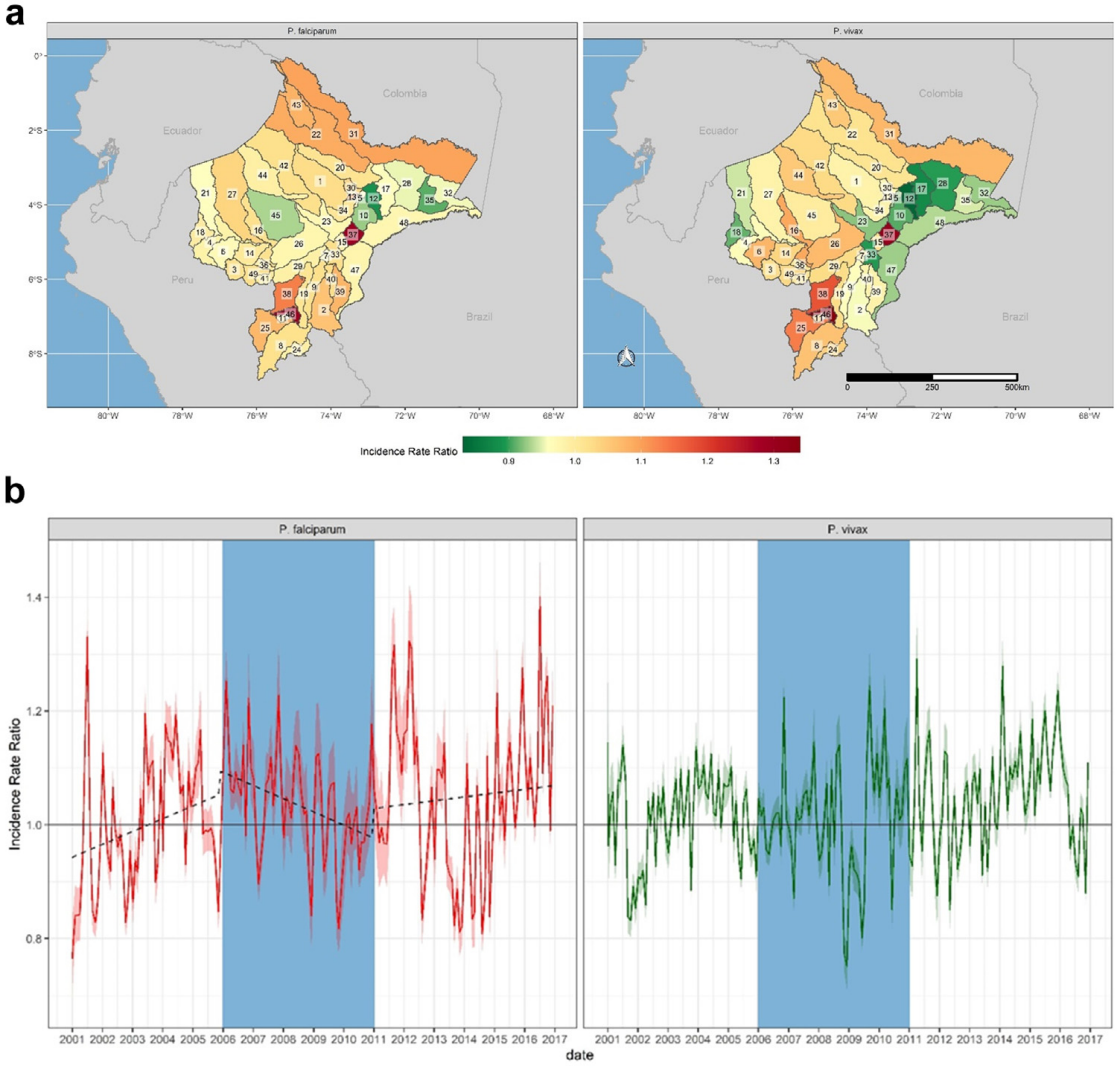
**Spatial and Temporal effects of minimum temperature on malaria incidence rates across Loreto.** District-level **(a)** and monthly **(b)** effects of minimum temperature (degrees Celsius) on *P. falciparum* (left) and *P. vivax* (right). The dotted line in the plot for P. falciparum in panel B shows increasing temporal effects before and after the PAMAFRO program, with falling incidence rates during the program. Incidence Rate Ratios are based on a 1-degree increase in minimum temperature. District labels are as follows: 1) Alto Nanay, 2) Alto Tapiche, 3) Balsapuerto, 4) Barranca, 5) Belen, 6) Cahuapanas, 7) Capelo, 8) Contamana, 9) Emilio San Martin, 10) Fernando Lores, 11) Inahuaya, 12) Indiana, 13) Iquitos, 14) Jeberos, 15) Jenaro Herrera, 16) Lagunas, 17) Las Amazonas, 18) Manseriche, 19) Maquia, 20) Mazan, 21) Morona, 22) Napo, 23) Nauta, 24) Padre Marquez, 25) Pampa Hermosa, 26) Parinari, 27) Pastaza, 28) Pebas, 29) Puinahua, 30) Punchana, 31) Putumayo, 32) Ramon Castilla, 33) Requena, 34) San Juan Bautista, 35) San Pablo, 36) Santa Cruz, 37) Saquena, 38) Sarayacu, 39) Soplin, 40) Tapiche, 41) Teniente Cesar Lopez Rojas, 42) Tigre, 43) Torres Causana, 44) Trompeteros, 45) Urarinas, 46) Vargas Guerra, 47) Yaquerana, 48) Yavari, 49) Yurimaguas.

**Fig. 6:**
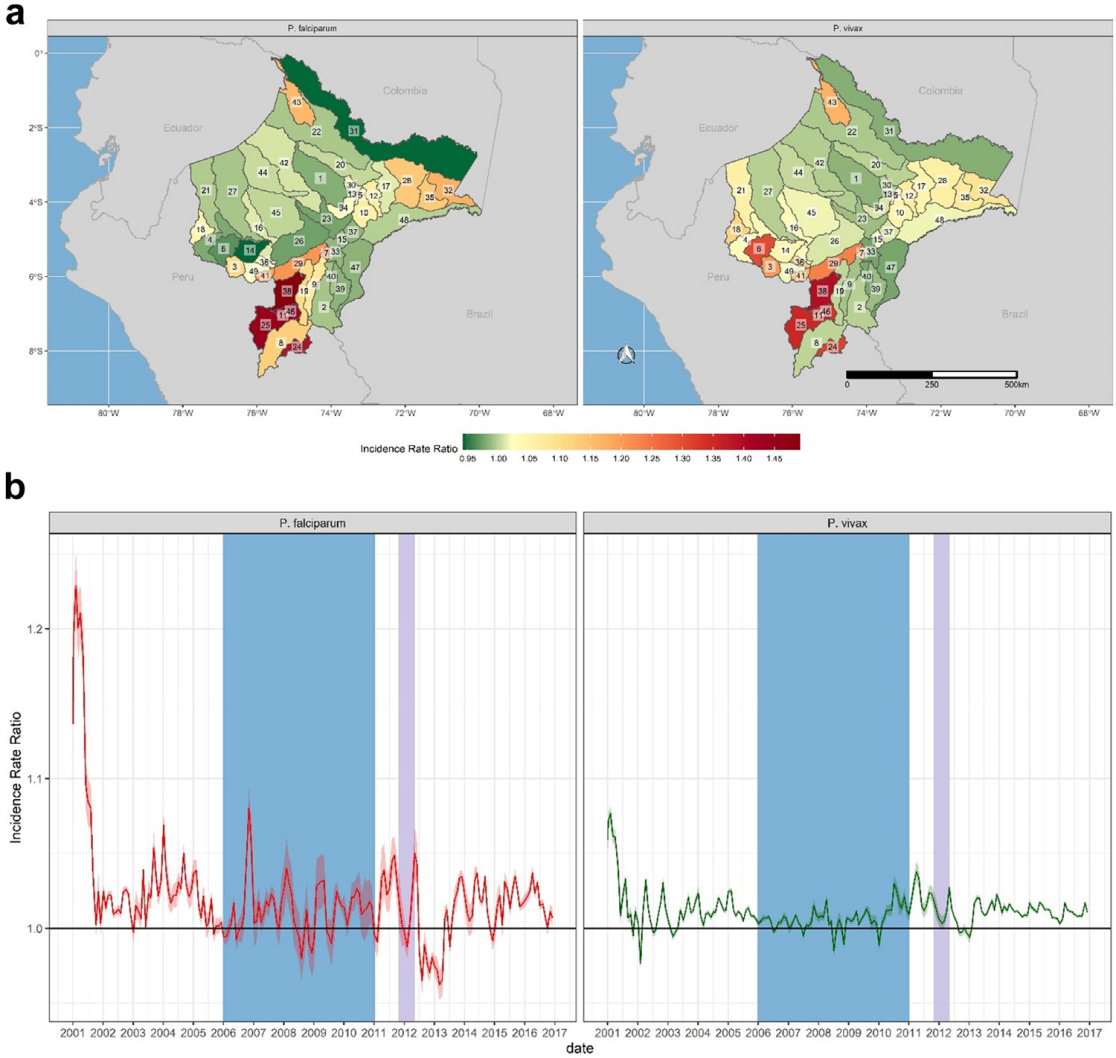
**Spatial and temporal effects of spatio-temporally lagged cumulative *P. vivax* and *P. falciparum* incidence. (a)** District-level effect of spatio-temporally lagged cumulative incidence for *P. falciparum* (left) and *P. vivax* (right). Non-shaded districts are those where non-significant effects were observed. District labels are as follows: 1) Alto Nanay, 2) Alto Tapiche, 3) Balsapuerto, 4) Barranca, 5) Belen, 6) Cahuapanas, 7) Capelo, 8) Contamana, 9) Emilio San Martin, 10) Fernando Lores, 11) Inahuaya, 12) Indiana, 13) Iquitos, 14) Jeberos, 15) Jenaro Herrera, 16) Lagunas, 17) Las Amazonas, 18) Manseriche, 19) Maquia, 20) Mazan, 21) Morona, 22) Napo, 23) Nauta, 24) Padre Marquez, 25) Pampa Hermosa, 26) Parinari, 27) Pastaza, 28) Pebas, 29) Puinahua, 30) Punchana, 31) Putumayo, 32) Ramon Castilla, 33) Requena, 34) San Juan Bautista, 35) San Pablo, 36) Santa Cruz, 37) Saquena, 38) Sarayacu, 39) Soplin, 40) Tapiche, 41) Teniente Cesar Lopez Rojas, 42) Tigre, 43) Torres Causana, 44) Trompeteros, 45) Urarinas, 46) Vargas Guerra, 47) Yaquerana, 48) Yavari, 49) Yurimaguas. **(b)** Monthly temporal effect of spatio-temporally lagged cumulative incidence for *P. falciparum* (left) and *P. vivax* (right). For both panels, Incidence Rate Ratios are based on an increase of one case of *P. falciparum* or *P. vivax* across each district's neighbors per 1000 population at risk the previous month.

Minimum temperatures also increased across Loreto by an average of 0.5 °C over the study period, from an average of 23.3 degrees C in the first five years of the study period to 23.8 degrees C in the last five years (Supplemental Fig. S8a). As with rainfall, temperature varied over space and time, with the greatest effects generally observed in those districts with higher rates of temperature change (Supplemental Fig. S10). More broadly, districts along the Napo, Pastaza, and Tigre rivers, where much of the resurgence in malaria transmission was concentrated, significant increases in risk were associated with increasing temperatures (Fig. 5a). Conversely, in districts along the Amazon river, we observed null or negative associations between minimum temperature and malaria incidence, particularly for *P. vivax*. For *P. falciparum,* there was also an apparent decline in the association between minimum temperature and transmission during the PAMAFRO program, such that by the end of the program, the effect was largely null. This trend was reversed after the program's end (Fig. 5b).

Spatio-temporally lagged cumulative incidence rates also exhibited varying effects (Fig. 6) and were associated with increased transmission along the Amazon and Ucuyali river corridors, as well as along the borders with Brazil in the southeast and Ecuador in the northeast. The largest associations were observed in the far southwest of Loreto, where an increase of one case per 1000 population at risk among neighboring districts during the prior month was associated with increases in incidence rates as high as 12% and 39% for *P. vivax* (IRR 1.12, 95% UI 1.11–1.13) and *P. falciparum* (IRR 1.39, 95% UI 1.36–1.42), respectively. Collectively, these districts exhibited very low transmission for the duration of the study period, with a total of 282 *P. vivax* and 24 *P. falciparum* cases during the study period, many of which are likely imported from neighboring districts. Further, these districts had among the lowest levels of intervention coverage in the region.

Finally, interventions supported by the PAMAFRO program also appeared to diminish the effects of climatic and demographic risk factors for transmission. For example, 73% of the incidence rate ratios capturing the effect of spatio-temporally lagged *P. vivax* were significantly greater than one during the PAMAFRO period (48% for *P. falciparum*), compared to 90% in the pre-PAMAFRO period (83% for *P. falciparum*) and 91% in the post-PAMAFRO period (61% for *P. falciparum*). Additionally, the effects along the river corridors and border areas were diminished in districts that both received greater intervention support and were adjacent to districts receiving support, relative to those districts that received no intervention support. Similarly, 35% of the incidence rate ratios capturing the effect of cumulative rainfall on *P. vivax* transmission (27% for *P. falciparum*) were significantly greater than one during the PAMAFRO period, compared to 58% (47% for *P. falciparum*) prior to PAMAFRO. As noted, for much of the post-PAMAFRO period, cumulative rainfall was associated with lower incidence of transmission, with 17% of incidence rate ratios for *P. vivax* (29% for *P. falciparum*) being significantly greater than one. The effects of minimum temperature on *P. vivax* were also more often associated with increased incidence rates before (45%) and after (61%) the PAMAFRO program than during (38% for *P. vivax*). Finally, cumulative rainfall was more frequently associated with increased transmission during the period prior to PAMAFRO (47% for *P. falciparum*; 58% for *P. vivax*), decreasing to 25% (*P. falciparum*) and 35% (*P. vivax*) during the program.

## Discussion

This study highlights the success of the PAMAFRO program in Peru, and provides spatially-explicit and intervention-specific estimates of the effects of interventions on *P. vivax* and *P. falciparum* incidence, while also accounting for important environmental and demographic confounders, and allowing for spillover effects.^7^ These estimates can be used to guide decision-making around what interventions to deploy and in which districts to optimize control, which is particularly important given the Global Fund's withdrawal of funding in much of the region. For example, across all interventions, we did not observe protective effects where an intervention was not deployed, indicating that spillover effects from neighboring districts cannot alone reduce transmission. Rather, they can enhance intervention effectiveness where they are deployed, suggesting that maximizing spatial coverage may maximize effectiveness.

Our findings also highlight some of the prevailing risk factors for transmission in the region, which were not able to be evaluated in the prior studies. For example, interventions such as strengthening diagnostics and training health workers demonstrated largely null effects, or were associated with increased transmission. One potential reason is that these interventions lead to increased case detection, which are critical to understanding the true underlying incidence in a district such that necessary resources to treat and prevent malaria can be deployed. These interventions appeared to be least effective along border regions and some river corridors, which suggests increased case detection in hard-to-reach areas or among hard-to-reach populations, such as migrants, a population that must be reached for successful malaria control and elimination. Indeed, our findings indicate that the effects of spatio-temporally lagged malaria incidence are highest along river systems, border areas, and low transmission settings, where human mobility can sustain malaria transmission at low levels. Coupled with our observation that interventions are most effective in districts that are adjacent to districts that also received interventions, our results support the need for malaria control programs to coordinate across districts, as well as with the surrounding countries of Brazil, Colombia, and Ecuador.

The post-PAMAFRO resurgence in transmission was driven by a number of factors, including increasing climate variability. Over the duration of the study period, minimum temperatures increased by an average of 0.5 °C across the region. These increases varied over space, and our findings show that the effects of minimum temperature on transmission were greatest in areas with the highest rates of temperature change. The region also experienced increasing variability and intensity of rainfall events. Similar to temperature, the effects of rainfall were also most severe in areas with the greatest increases in rainfall. In the short term, extreme rainfall events can cause severe flooding and population displacement. An example of this was the severe flooding across Loreto in 2011 and 2012 led to large-scale population displacement and damaged or destroyed over 72,000 households, 1724 schools, and 54 health centers.^29^ Paradoxically, much of the post-PAMAFRO period is characterized by protective effects of rainfall. A number of factors may account for this. First, population displacement and the destruction of health infrastructure may explain the reduced incidence rate ratios. Additional support for this hypothesis comes from the effect of spatio-temporally lagged incidence rates, with the effect increasing over time starting towards the end of the PAMAFRO program when rainfall began its dramatic increase and peaking at the end of the flood period, consistent with prior observations that malaria incidence peaks at the end of the rainy season, and suggesting that cases may have been diagnosed in areas where people settled following initial displacement and the recession of flood waters. Conversely, extreme rainfall can wash away vector larvae or otherwise disrupted, previously stable aquatic habitats, suggesting that extreme rainfall may exhibit protection in the absence of population displacement or other factors that place populations at increased risk. Nevertheless, these effects are extremely difficult to disentangle given that disruptions due to flooding can disrupt the surveillance system and case reporting owing to the displacement of staff and destruction of other infrastructure, which is unmeasured here and may have remained in place had the flooding not occurred. Another related driver of the malaria resurgence may be liberal extractive resource policies that are causing more rapid land clearing and labor migration that is related to malaria, which can produce habitats favorable to *Ny. darlingi* mosquito populations and increase contact between vectors and humans.^30^^,^^31^

While our results show that climate change and its effects are detrimental to malaria control, we also found that sustained intervention support can offset the effects of climate change. Specifically, we showed that the interventions deployed can diminish climate effects on transmission region wide. This has implications for malaria control in the region and highlights the ongoing need for continued interventions to offset increases in risk owing to changing climate conditions.

Our work here builds on a prior analysis of meteorological effects on malaria transmission in the presence of interrupted malaria control in Loreto conducted by Carrasco-Escobar and colleagues.^13^ Specifically, their analysis investigated time-varying effects of minimum temperature and rainfall (among other variables) on monthly malaria transmission, and observed significant reductions in incidence associated with increased rainfall in the latter half of the PAMAFRO period and for much of the post-PAMAFRO period, whereas our analysis observed these reductions principally in the post-PAMAFRO period. There were similar differences in our analyses of temperature. A number of factors may account for this. First, our measures of environmental covariates are made at the weekly scale, using different meteorological data, with effects that vary monthly. Second, our inferential objectives were to estimate intervention- and district-specific impacts of the PAMAFRO program, while adjusting for meteorological variables. Third, we allow the effects of environmental covariates vary over both space and time. Finally, we include different sets of other covariates.

The success of PAMAFRO resulted in its being named the inaugural Malaria Champion of the Americas, an annual honor bestowed by PAHO since 2009.^32^ PAMAFRO's success has been attributed to engagement of communities to prevent and control malaria.^33^ The program used a participatory approach that fused malaria education into the national education curricula and created collective incentives between four Andean nations to work together on malaria control. In 2009, the last comprehensive PAMAFRO program report highlighted the need for continued engagement with communities, continued education, strengthening monitoring and evaluation, and, most importantly, institutionalization of programs into Ministries of Health and continued funding to maintain human technical capacity.^34^ By 2010, there was already a recognized need to train local microscopists to maintain community health system strength.^34^ However, it is apparent that PAMAFRO investments were withdrawn without a clear plan for continuity, resulting in malaria incidence returning to pre-intervention levels within a few years.

The abrupt end of PAMAFRO support, lack of a sustainability plan, and the subsequent resurgence of malaria in the Amazon serves to inform current global programs attempting to taper support of disease control.^35^ Addressing these concerns is imperative given a review of malaria resurgence events, which found that 91% of such events were due in part to the weakening of malaria control programs, highlighting the urgent need to address the challenges in maintaining financing for malaria control in the presence of declining or low transmission.^36^

Our study has important limitations. First, data are from passive surveillance. While the Ministry of Health occasionally conducts active surveillance in response to observed increases in transmission, cases detected by active surveillance are not incorporated into the surveillance system, indicating an undercount of the true case load. For example, the vast majority of malaria cases in the region are asymptomatic, and estimates from Peru suggest that only roughly 45% of cases were detected by the surveillance system in 2016, the final year of the study period.^6^

Second, our intervention data only includes information on where and when interventions occurred, and not the degree of intervention coverage (e.g. number of bed nets distributed). Third, data do not cover settings outside Loreto where the PAMAFRO program existed. As a result, we do not know the program's impact across the region, although malaria transmission declined in every place where the program was implemented. Unfortunately, the data to evaluate intervention- and spatially-explicit effects in these regions are not currently available, precluding us from extending our evaluation to these settings. Additionally, Loreto is ecologically similar to the broader region, and WHO reporting indicates that other countries also experienced substantial declines followed by a resurgence. For example, published data on cases in other PAMAFRO regions in Colombia show declines from 22,472 in 2005 to 12,382 in 2008; in Ecuador, cases fell from 6245 to 2053 during this same period. In Venezuela, total cases fell from 7351 to 6019.^33^^,^^37^ Thus, our findings are likely similar to what occurred in other countries that received the intervention.^38^ On the other hand, malaria transmission continued to decrease in Atalaia do Norte, which borders the Yavari district in Peru, even after the discontinuation of the Global Fund Program in Brazil after 2013.^39^

A fourth limitation to our study is that, while our analysis includes spatio-temporally lagged cumulative incidence as a covariate meant to capture the effects of human mobility on malaria spread, we do not measure migration directly nor account for cross-border malaria importation.^40^^,^^41^ Migration in the Amazon is characterized generally as short-term (i.e., visiting family, markets, short-term labor), seasonal (i.e., employment, education), and permanent, variable by age and sex.^42^ Other work has investigated the effects of human mobility on malaria transmission in the Loreto region using a variety of methods, including travel surveys, GPS data loggers, and entomological data. Findings from this work suggest that human mobility driven by work, trading, or visiting family can sustain malaria transmission among the riverine communities that dominate the region, in part because mobile populations come into contact with vectors in the absence of vector control.^43^, ^44^, ^45^ Further, molecular work in the region has shown a high degree of malaria gene flow or population differentiation, each of which is related to levels of human mobility between locations.^46^, ^47^, ^48^ Our work builds on these studies by constructing a malaria-relevant measure of mobility for each district in Loreto, and allowing its effects on transmission to vary over space and time. Nevertheless, a key driver of the post-PAMAFRO resurgence of malaria in the region has been the political instability in Venezuela,^49^, ^50^, ^51^ which has displaced over six million people according to the International Organization for Migration, 80 percent of whom are residing in other Latin American countries, where the overwhelming majority also fail to receive adequate social, economic, and medical care.^52^ Data on the size and locations of the displaced population in Peru is still lacking, as is the number of imported of malaria infections into Peru from Venezuela. As a result, we cannot quantify their effects on the post-PAMAFRO resurgence.

Finally, we do not incorporate entomological data into our analysis, meaning we are missing a key population (i.e. mosquitos) that influences heterogeneity in transmission, and is itself responsive to malaria control. For example, an entomological study conducted in 2011–12, following the end of the PAMAFRO program, found increased rates of outdoor biting among *Anopheles darlingi* mosquitoes.^53^ A subsequent study conducted between 2013 and 2015 found that this trend had reversed, with increased rates of indoor biting relative to outdoor biting.^25^ No additional LLIN distributions had been conducted between studies, suggesting that LLIN interventions had changed the biting behavior of *An. darlingi* mosquitos, which then reverted to indoor biting behavior as LLINs aged. Another study found evidence of population replacement between 2006 and 2012–14, concurrent to the LLIN distributions implemented by PAMAFRO. Since these studies, more recent work in the region has again found increased outdoor biting behavior among *An. darlingi* mosquitoes, possibly in response to renewed efforts to control malaria in the region.^54^^,^^55^

Despite these limitations, we have provided evidence on the effects of different malaria interventions across a malaria-endemic region of Latin America, how the effects of those interventions varied spatially and by malaria species, and how they buffered the effects of other prevailing environmental and demographic risk factors for transmission. Our findings build on existing evidence that the biggest threat to malaria resurgence is the weakening of malaria control, largely due to inadequate financing.^36^

## Contributors

MMJ, AGL, and WKP conceptualized the project. GCR and BFZ were responsible for compiling environmental estimates. AGL and GSM facilitated access to the malaria surveillance data. MMJ processed the data and conducted the statistical analysis. MMJ, GCR, CPD, GSM, AFB, AGL, BFZ, and WKP interpreted the results. MMJ and WKP wrote the manuscript. All authors approved the final version of the manuscript.

## Data sharing statement

Malaria surveillance data is publicly available from CDC-Peru (http://www.dge.gob.pe/salasituacional/sala/index/6_mapaCanal/87). All other data are available from the corresponding authors upon reasonable request.

## Editor note

The Lancet Group takes a neutral position with respect to territorial claims in published maps and institutional affiliations.

## Declaration of interests

We declare no conflicts of interest. WKP is a member of the Science Advisory Board for the Institute for Malaria and Climate Solutions.

## Appendix A Supplementary data

Supplementary Statistical Methods, Figs. S1–S11, Tables S1–S5 and STROBE statement
