# Supplementary material for: The impact of sustained malaria control in the Loreto region of Peru: a retrospective, observational, spatially-varying interrupted time series analysis of the PAMAFRO program
Source: Lancet Reg Health Am. 2023 Mar 16;20:100477. doi: 10.1016/j.lana.2023.100477 (PMC10036736; doi:10.1016/j.lana.2023.100477)
Supplement: Supplementary Statistical Methods, Figs. S1–S11, Tables S1–S5 and STROBE statement [file mmc1.docx]

**Supplementary Material**

# Statistical Methods

Our statistical model adopts an interrupted time series approach, which has the following general form:

$$y_{t}= \beta_{0}+ \beta_{1}*T+ \beta_{2}X+ \beta_{3}XT+\epsilon_{t}$$

where $y_{t}$ is the outcome of interest, in our case *P. falciparum* or *P. vivax* malaria during epidemiological week $t$, $T$ is a continuous covariate for time (epidemiological week), $X$ is a binary indicator for the intervention, $XT$ is an interaction between time and the intervention indicator, and $\epsilon_{t}$ is an error term. Under this basic specification, the model for $y_{t}$ prior to the intervention period is given by:

$$y_{t}= \beta_{0}+ \beta_{1}*T+ \epsilon_{t}$$

where the model merely captures the linear time trend in the outcome prior to the intervention taking effect. When the intervention takes effect, the model allows for a change in the intercept through the effect of $\beta_{2}$ as well as for a change in the slope through the effect of $\beta_{3}$.

We make a number of modifications to this basic formulation of an interrupted time series model, as follows. First, because the outcome data are counts of *P. falciparum* and *P. vivax* cases, we consider a Poisson likelihood to model the incidence rates. Second, because of the time needed to scale interventions sufficiently to have a population-level effect on malaria transmission, we remove the indicator $X$ from the model, but retain the $XT$ term (denoted $x$, below), thereby allowing for a change in slope only. Third, we include terms for each of the four interventions that made up the PAMAFRO program. Fourth, we lag the interventions by a year to allow them to be scaled and take effect. Fifth, because the interventions were initiated at different times in different places, and because of potential spillover effects, we model all districts together (as opposed to modelling univariate time series) and allow the intervention effects to vary spatially by assigning each intervention a conditionally autoregressive (CAR) prior distribution. Sixth, because malaria transmission exhibits strong seasonality due to variability in environmental factors, we allow the cumulative rainfall, minimum temperature, and spatio-temporally lagged incidence covariates to vary spatially and temporally (to account for the unobserved vector population and changing environmental conditions over the time series). Seventh, to further address seasonality, we include an exchangeable random effect for month. The model for a single district $s$ during a single epidemiological week $t$ in month $m$ can thus be written as:

$y_{s\left[ t \right]}=T_{\left[ t \right]}^{1}\alpha_{s}^{1}+T_{\left[ t \right]}^{2}\alpha_{s}^{2}+ \sum_{j=1}^{4} \boldsymbol{x}_{s\left[ t \right],j}^{T} \beta_{s,j}\boldsymbol{+}\sum_{k=1}^{3} V_{s\left[ t \right],k}^{T}\eta_{s,k}+ \sum_{k=1}^{3} V_{s\left[ t \right],k}^{T}\eta_{t,k}+I_{s\left[ t \right]}\gamma+ \theta_{m}$

where $y_{s\left[ t \right]}$ is the number of malaria cases (separately for *P. vivax* and *P. falciparum*) in district $s$ at time $t$, $\boldsymbol{x}_{s\left[ t \right]}^{T}$ is a $1\times4$ vector of interventions. The spatially-varying regression coefficients $\beta$ link the interventions covariates to the response via a log. The $1\times3$ vector $V_{s\left[ t \right],k}^{T}$ is a vector of covariates, each of which is allowed to vary spatially through $\eta_{s}$ and over time through $\eta_{t}$ using penalized splines. The variables $T_{\left[ t \right]}^{1}$ and $T_{\left[ t \right]}^{2}$ are covariates that sequentially count the number of epiweeks prior to the beginning of PAMAFRO ($T_{\left[ t \right]}^{1}$) and time since the PAMAFRO program ended ($T_{\left[ t \right]}^{2}$), with their effects varying spatially according to parameters $\alpha$. Finally, $I_{s\left[ t \right]}$ represents an indicator for the Iquitos district, with $\gamma$ its effect, while $\theta_{m}$ represents the random effect for month.

“We assign penalized complexity prior specifications for the hyperparameters governing the spatial processes, and use an improper CAR (ICAR) specification. We model the malaria outcome using the Poisson likelihood and include an offset of each district’s population, which we assume remains constant over the study period, consistent with prior analyses in the region.^1,2^ Model fit statistics are presented in Supplementary Table 1, showing that the lagging the interventions and assuming them to remain effective for an additional year yielded a better fit.”

**Supplemental Figures**

# Supplemental Figure 1

| **Supplemental Figure 1. Timing of long-lasting bed net distributions by district** |
| --- |
| 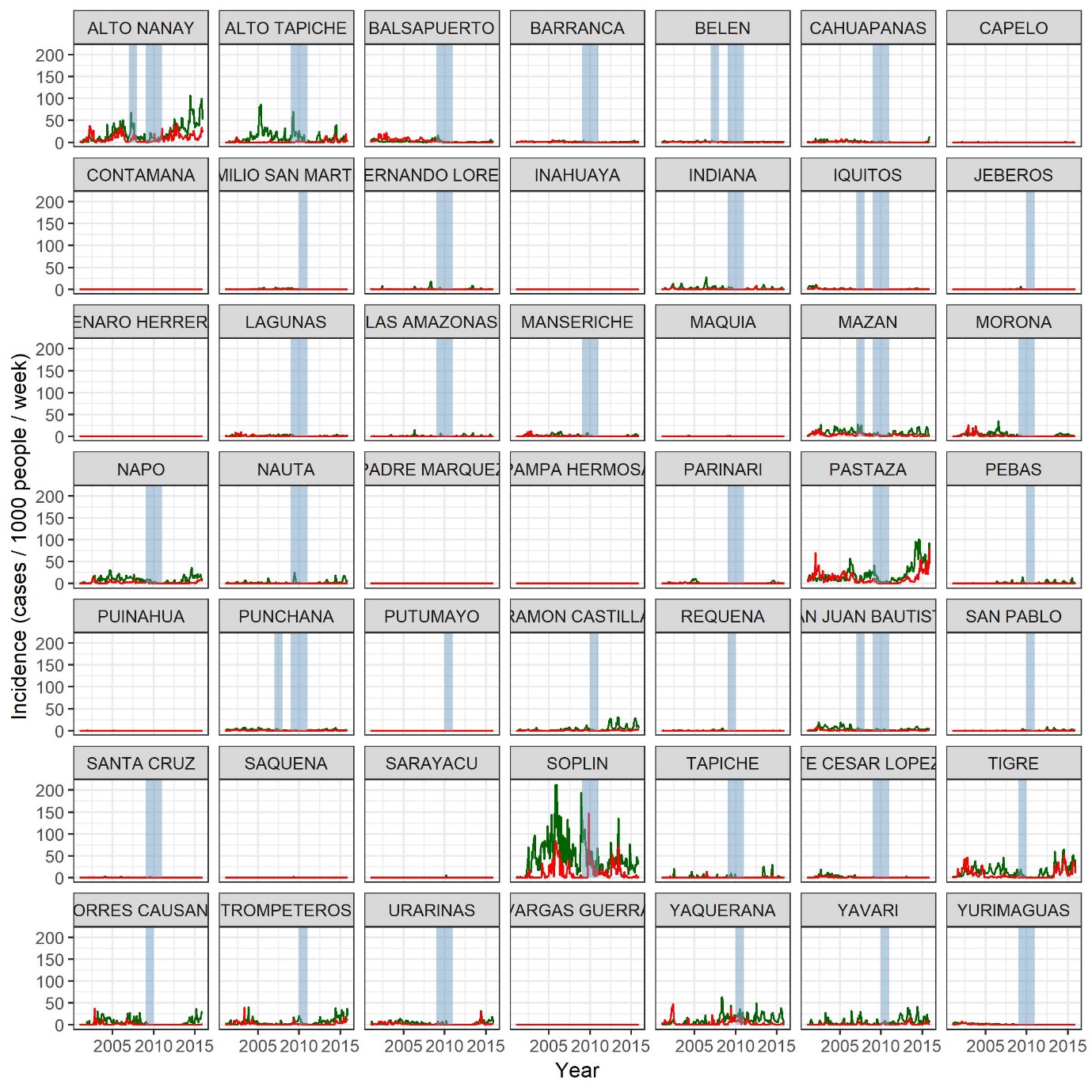 |

# Supplemental Figure 2

| **Supplemental Figure 2. Timing of environmental management campaigns by district** |
| --- |
| 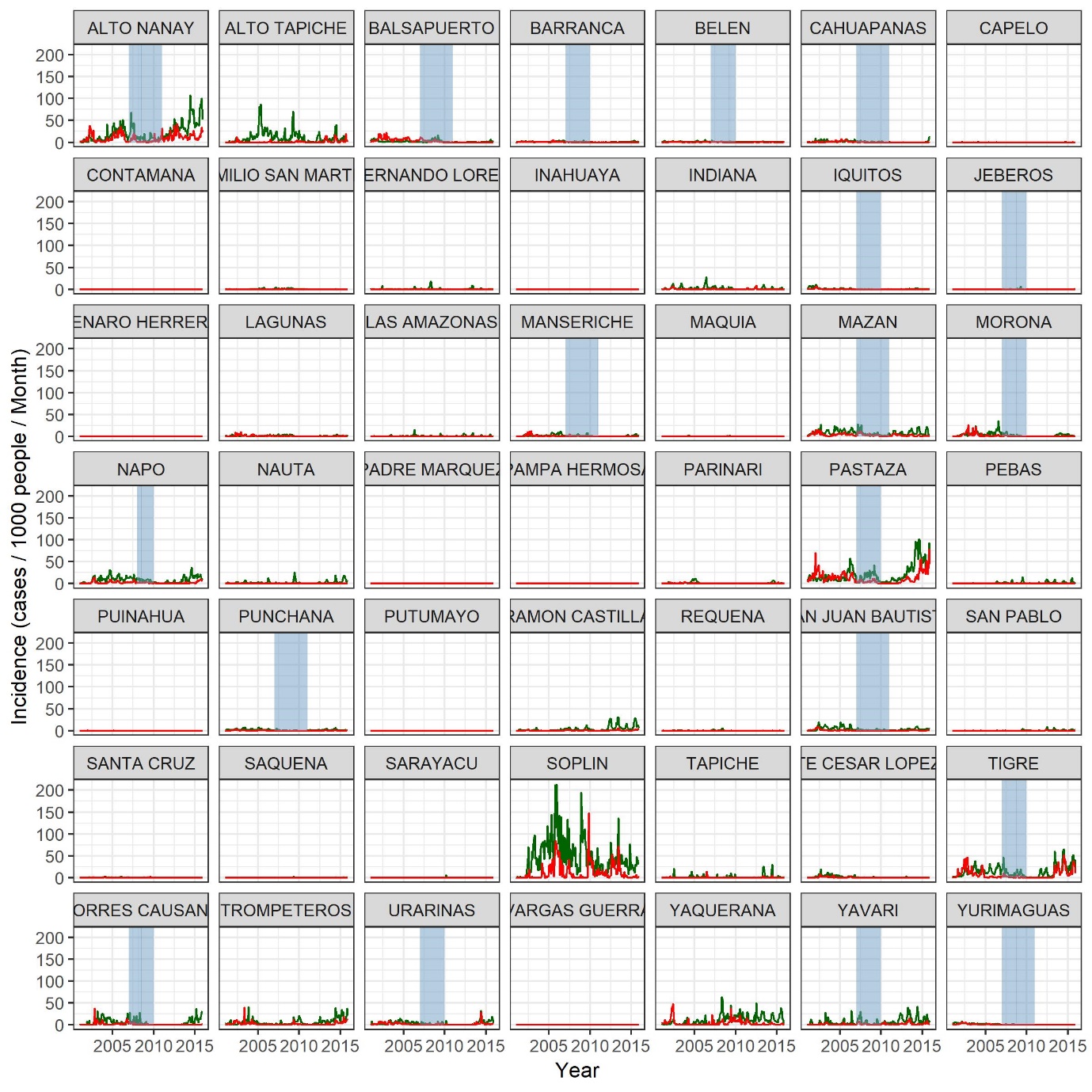 |

# Supplemental Figure 3

| **Supplemental Figure 3. Timing of strengthening diagnostic campaigns by district** |
| --- |
| 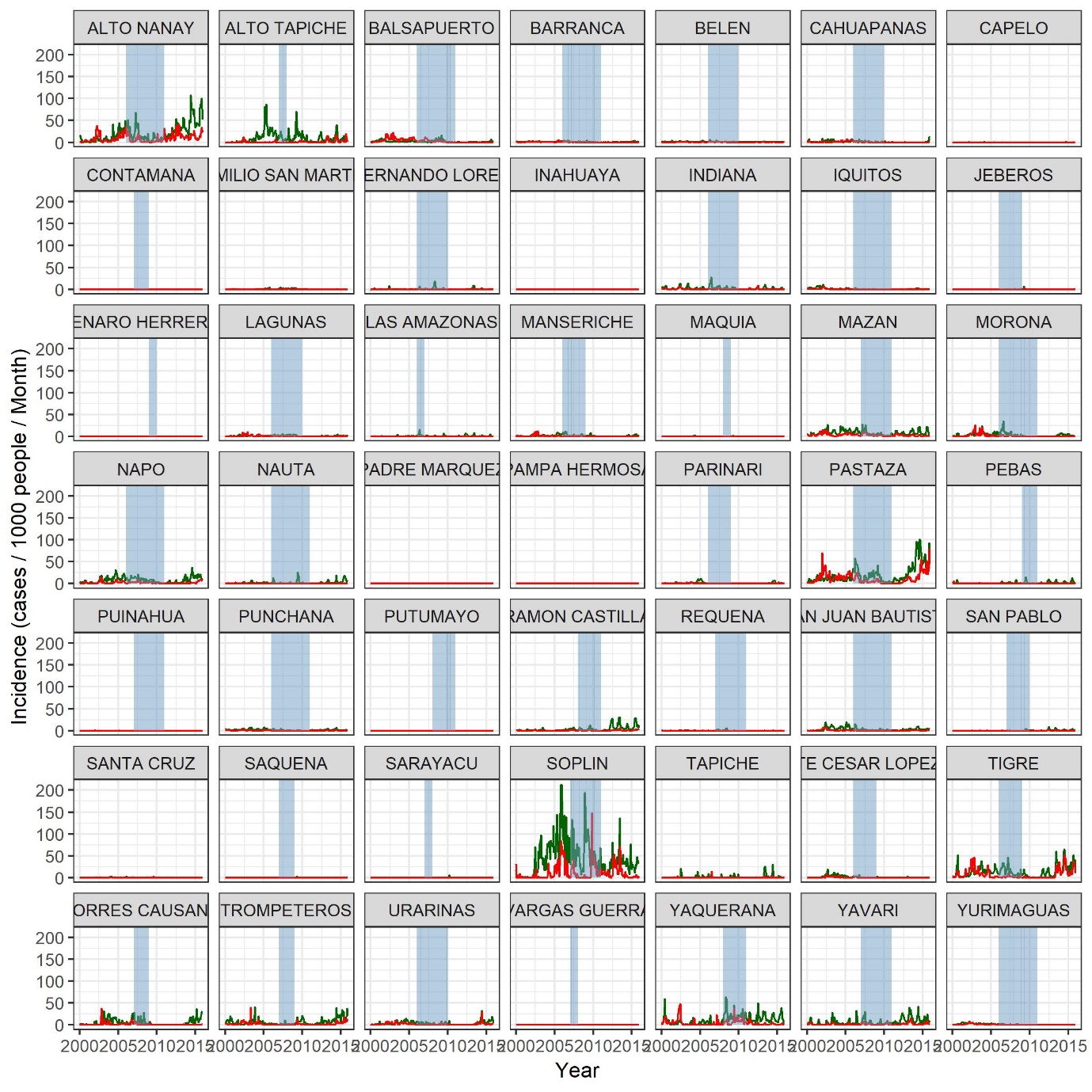 |

# Supplemental Figure 4

| **Supplemental Figure 4. Timing of health worker tranings by district** |
| --- |
| 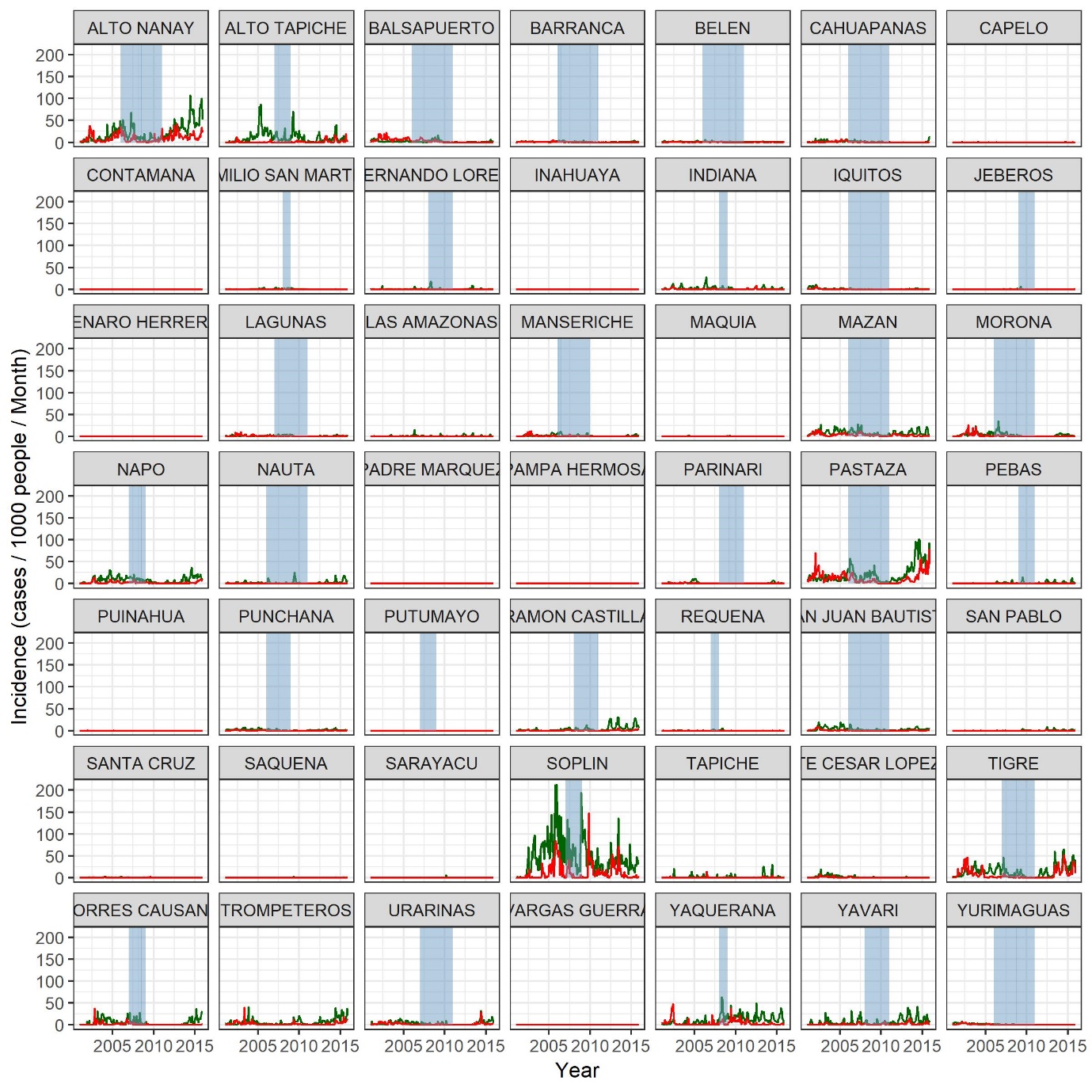 |

# Supplemental Figure 5

| **Supplemental Figure 5.** Average cumulative rainfall across the Loreto region of Peru. |
| --- |
| **A)**  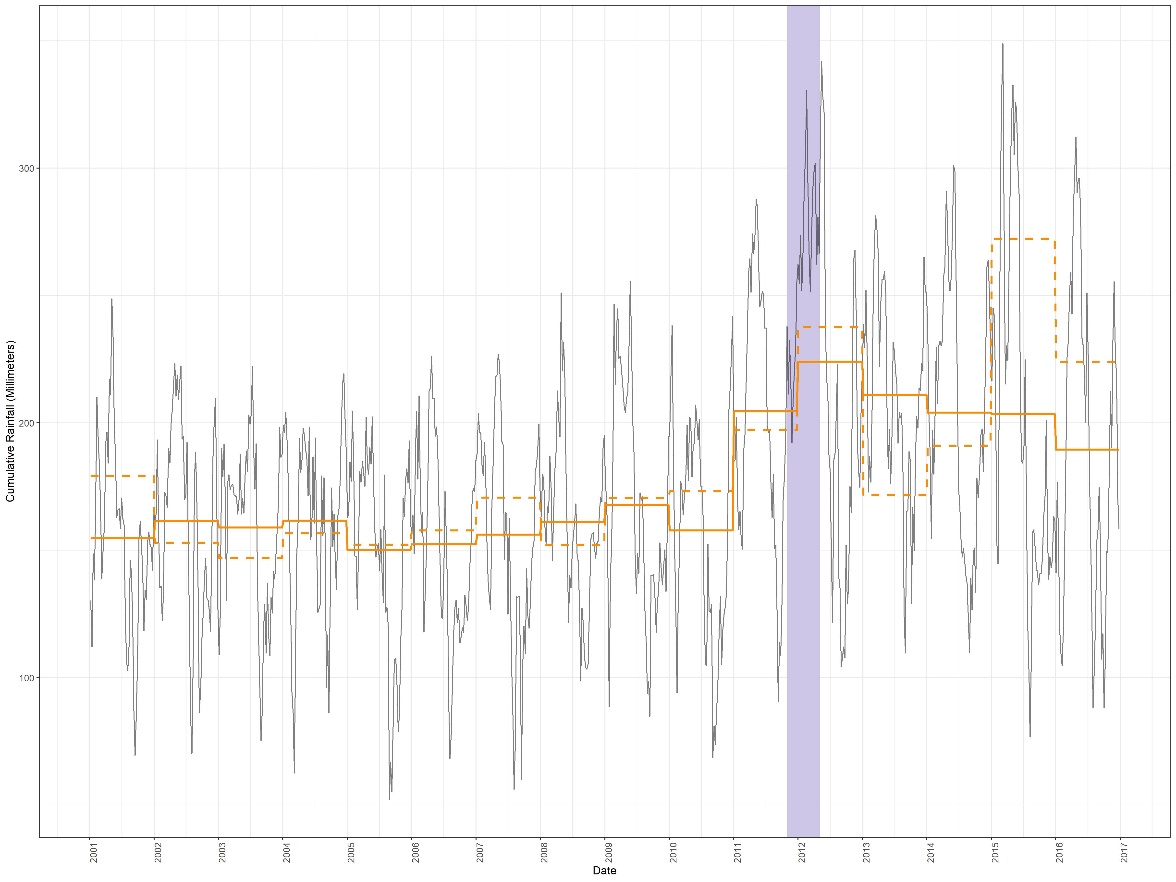 |
| **B)**  **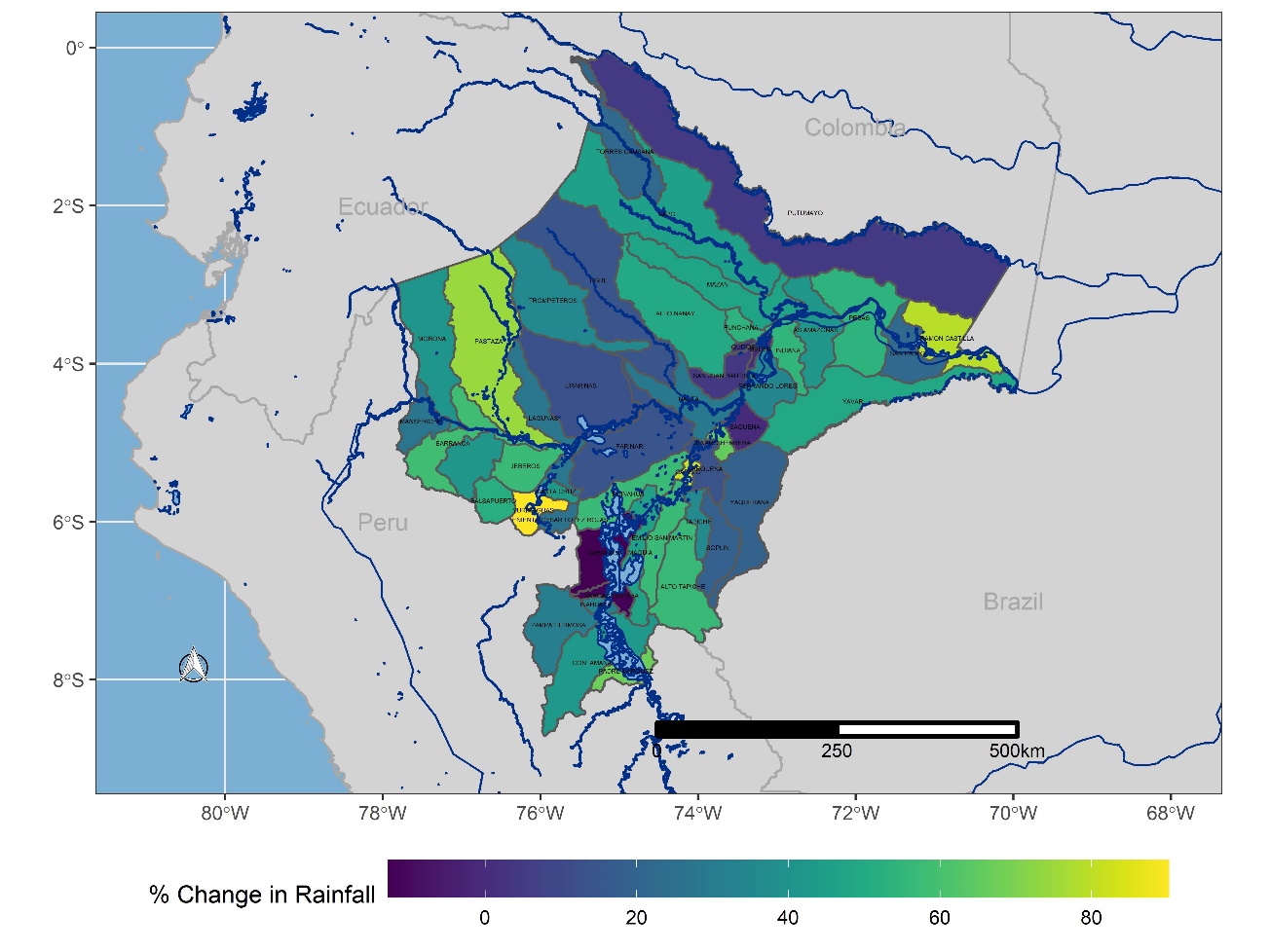** |
| **A)** Grey line incidates weekly cumulative rainfall for all Loreto. Solid orange line shows the yearly average, while the dotted orange line shows the range (maximum – minimum cumulative rainfall observed). The shaded region corresponds to the Loreto floods that occurred from November 2011 – April 2012, which resulted in substantial population discplacement. **B)** Percent change in average cumulative rainfall between the first five and the last five years years of the study period by district. |

# Supplemental Figure 6

| **Supplemental Figure 6.** |
| --- |
| 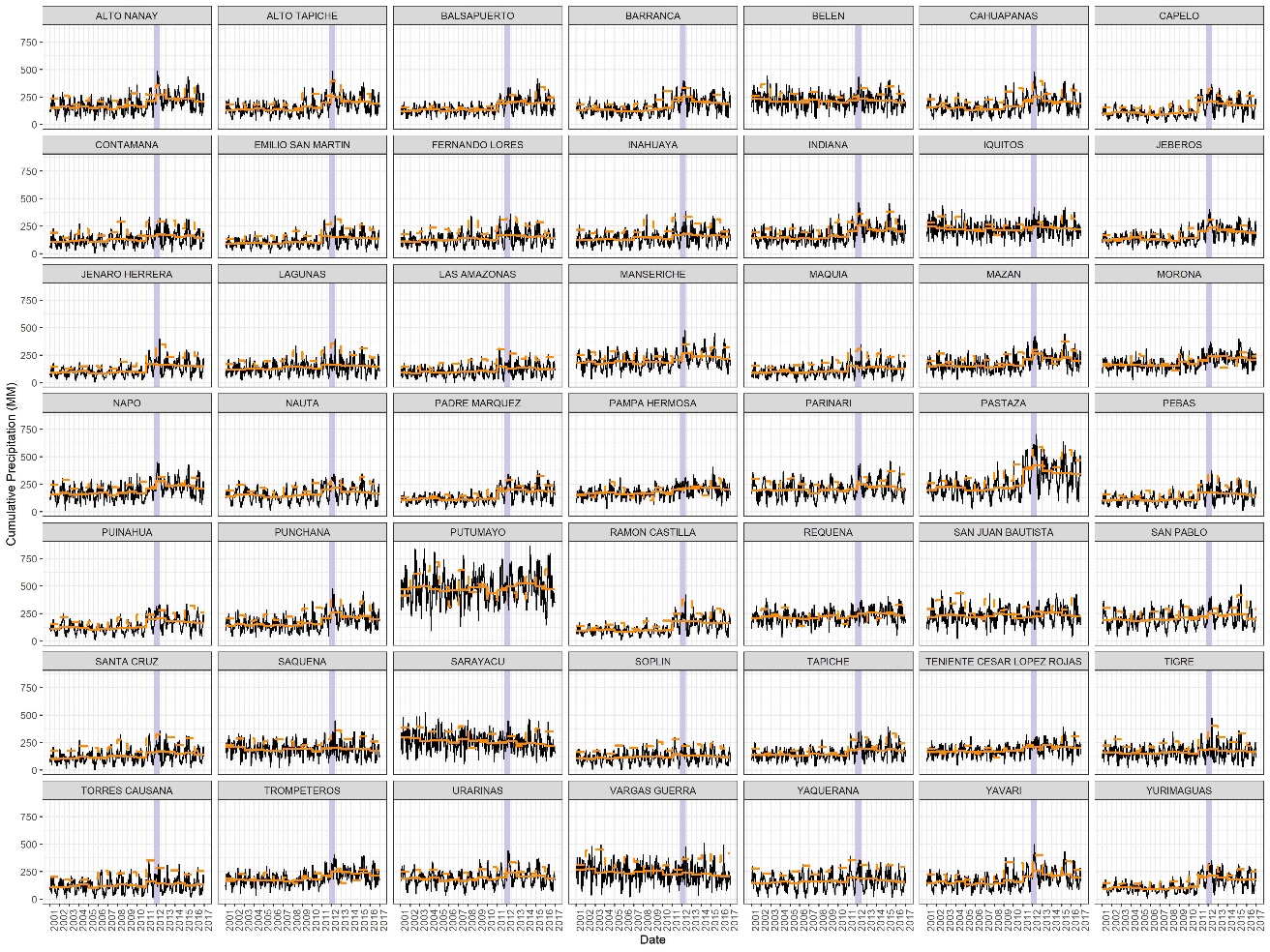 |
| Weekly estimates of cumulative rainfall in each district in Loreto spanning the study period. Orange line represents the annual average, while the dotted line represents the annual range (maximum – minimum). The shaded region corresponds to the Loreto floods that occurred from November 2011 – April 2012, which resulted in substantial population discplacement |

**Supplemental Figure 7**

| **Supplemental Figure 7.** The effect of rainfall on transmission in each district vs percent change in cumulative rainfall by district. |
| --- |
| 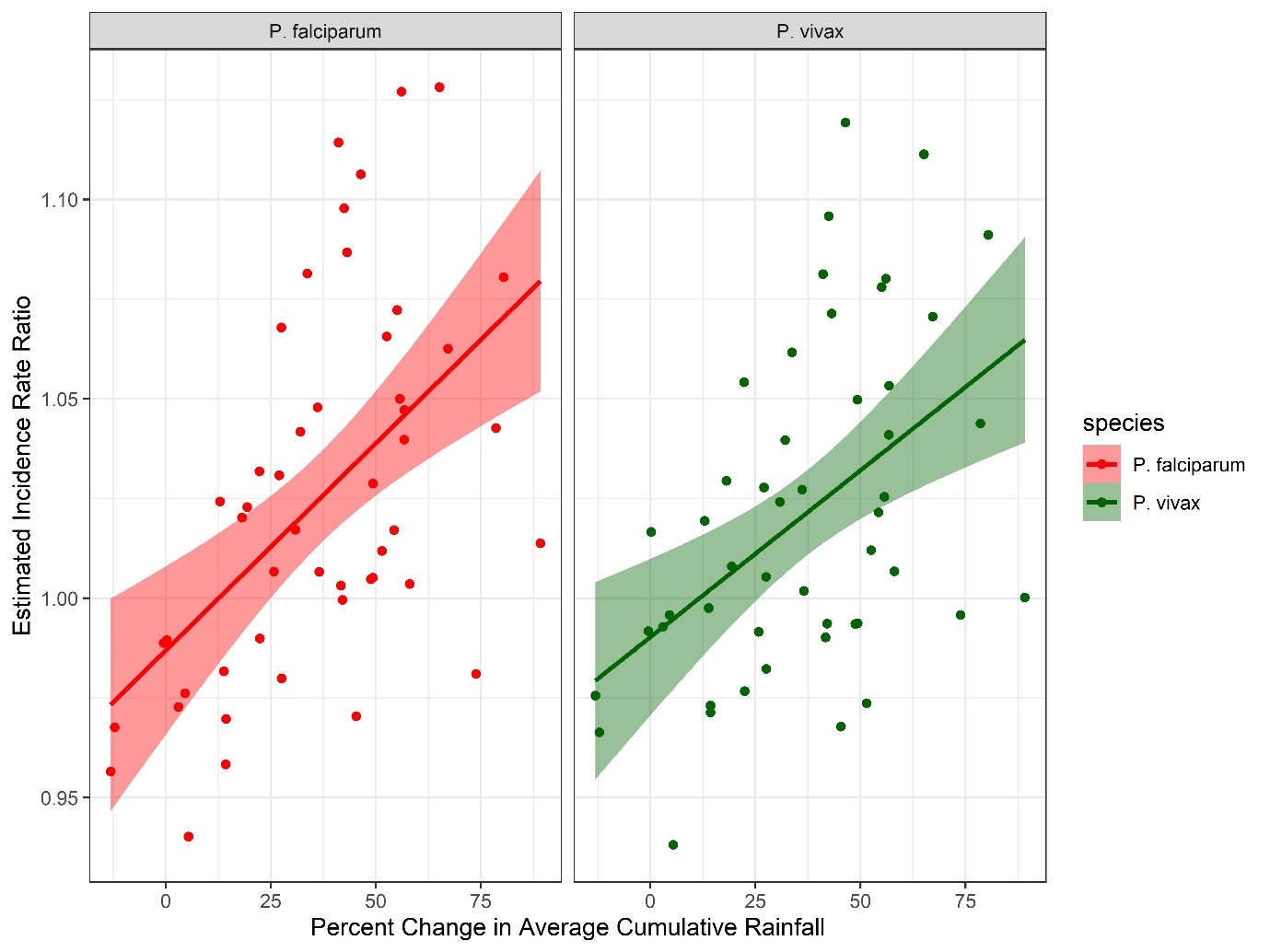 |
| The y-axis represents the estimated IRRs for the spatially-varying coeffienct capturing the effect of cumulative rainfall on *P. falciparum* and *P. vivax* transmission. The x-axis is the percent change in cumulative rainfall from the first five years of the study period to the last five years in each district. The solid line summarizes the linear regression between the estimated effect size and the percent change, with the shaded region corresponding to the 95% uncertainty interval. |

# Supplemental Figure 8

| **A)**  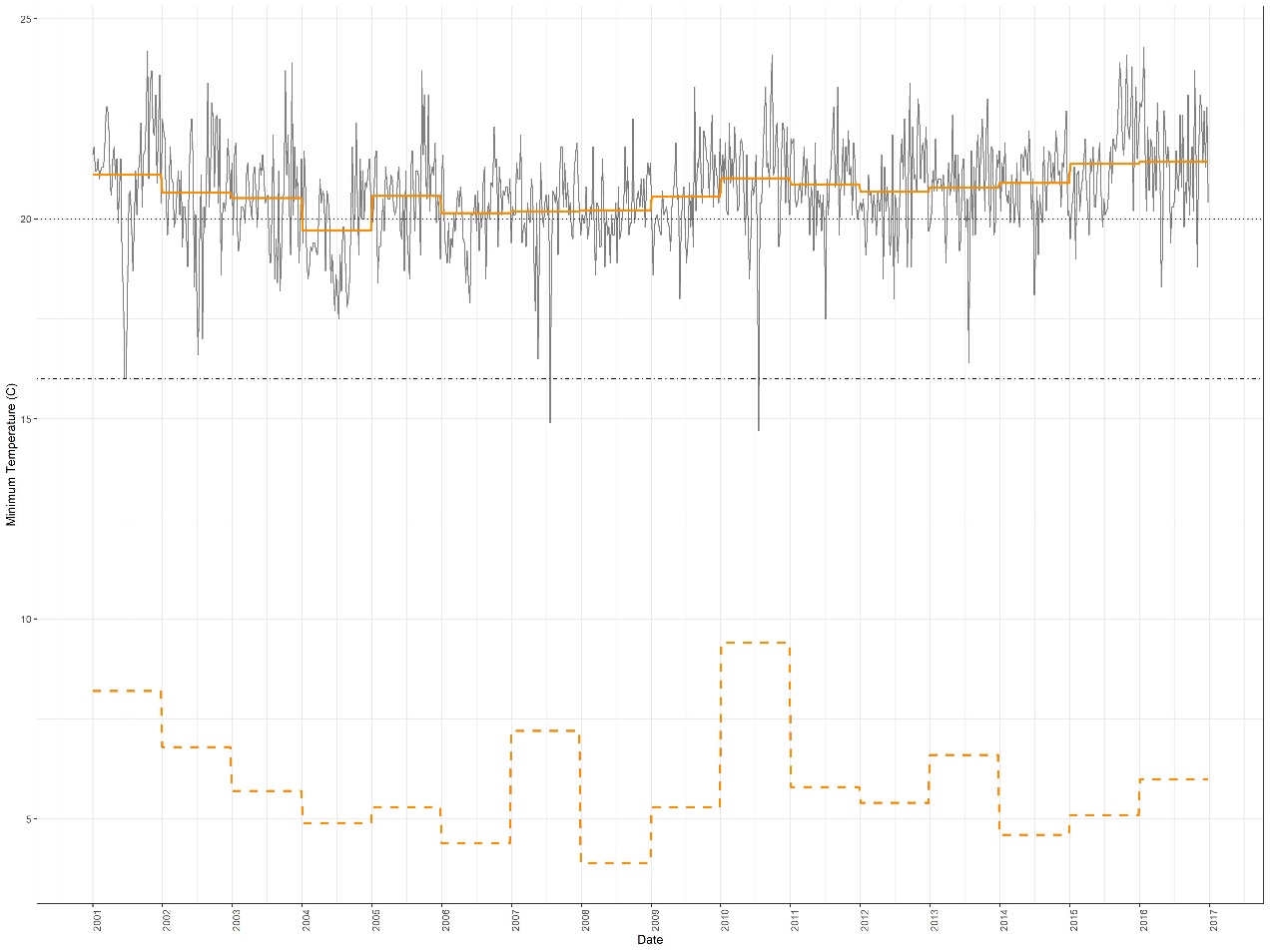 |
| --- |
| **B)**  **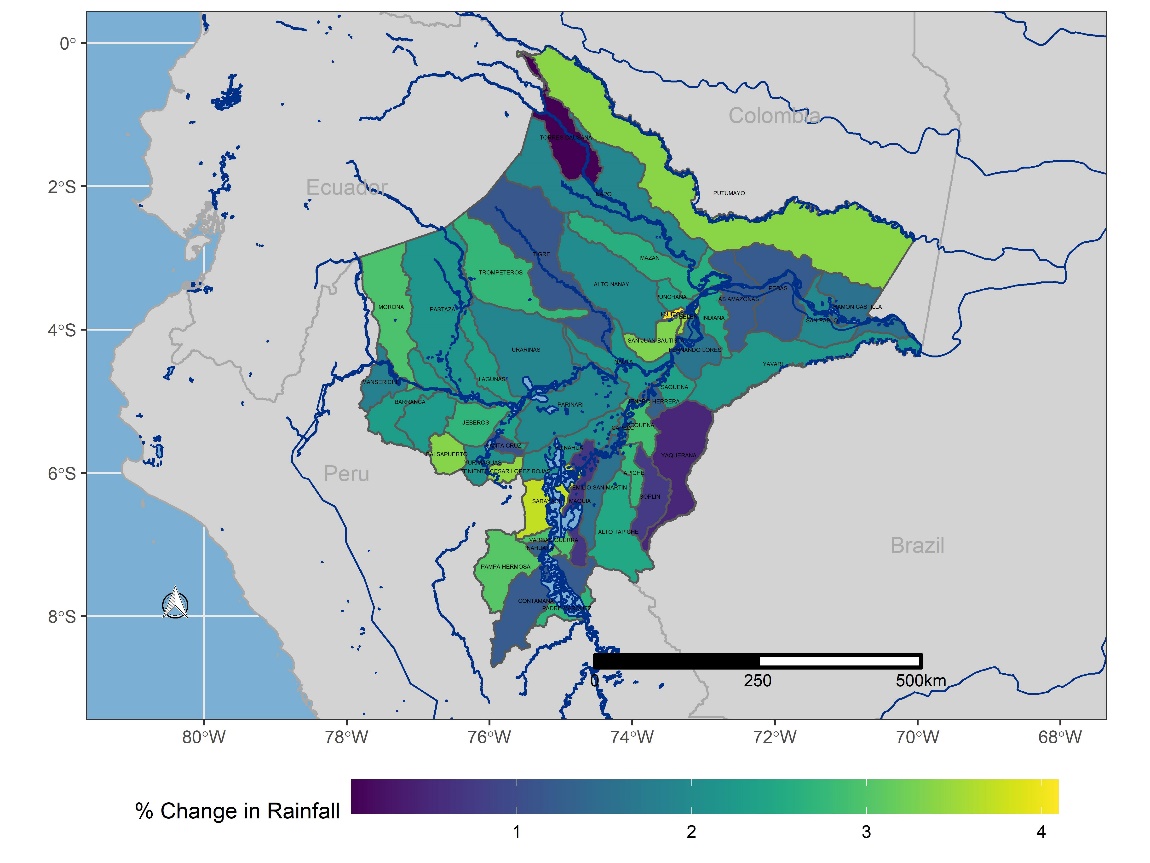** |

**(A)** Grey line incidates weekly minimum temperatures for all Loreto. Solid orange line shows the yearly average, while the dotted orange line shows the range (maximum – minimum). **(B)** Percent change in minimum temperature between the first five and the last five years years of the study period by district.

# Supplemental Figure 9

| Supplemental Figure 9. Weekly minimum temperature in degrees Celcius by district |
| --- |
| 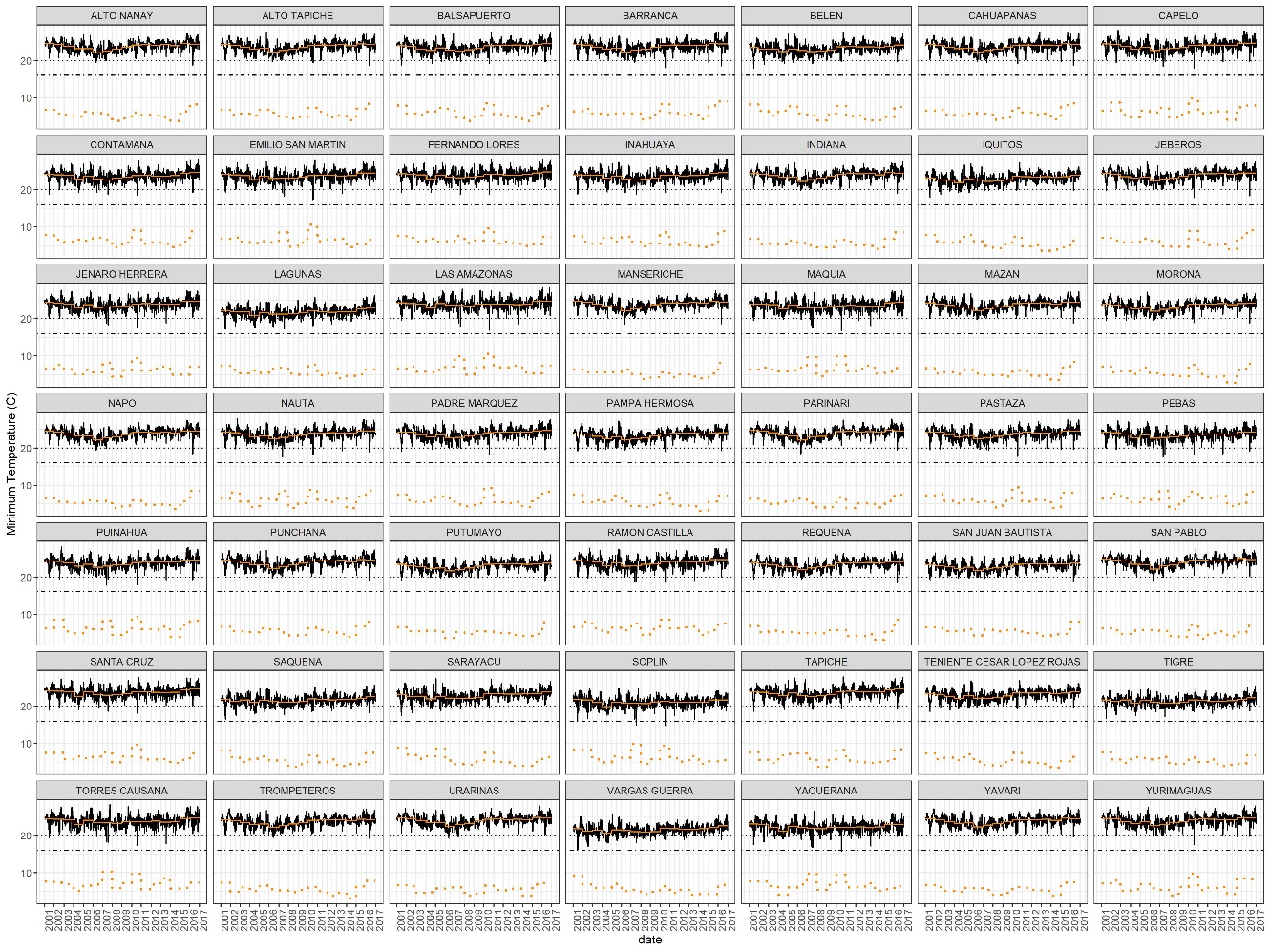 |
| Grey line incidates weekly minimum temperatures for each district. Solid orange line shows the yearly average, while the dotted orange line shows the range (maximum – minimum). |

# Supplemental Figure 10

| Supplemental Figure 10. Percent change in minimum temperature by district vs the effect of rainfall on transmission in each district |
| --- |
| 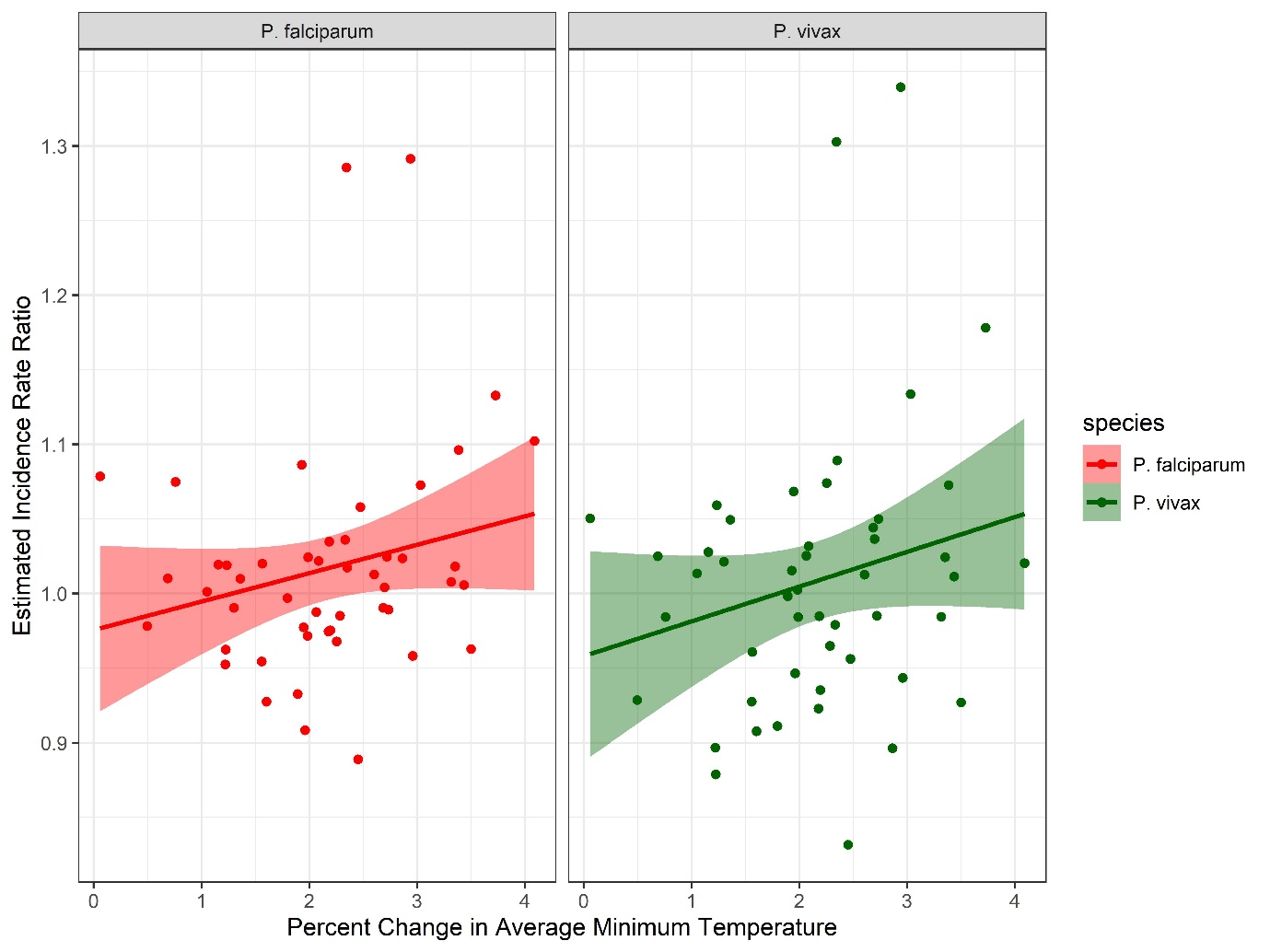 |
| The y-axis represents the estimated IRRs for the spatially-varying coeffienct capturing the effect of minimum temperature on *P. falciparum* and *P. vivax* transmission. The x-axis is the percent change in minimum temperature from the first five years of the study period to the last five years in each district. The solid line summarizes the linear regression between the estimated effect size and the percent change, with the shaded region corresponding to the 95% uncertainty interval. |

# Supplemental Figure 11

| **Supplemental Figure 11. Model performance by district over time.** |
| --- |
| 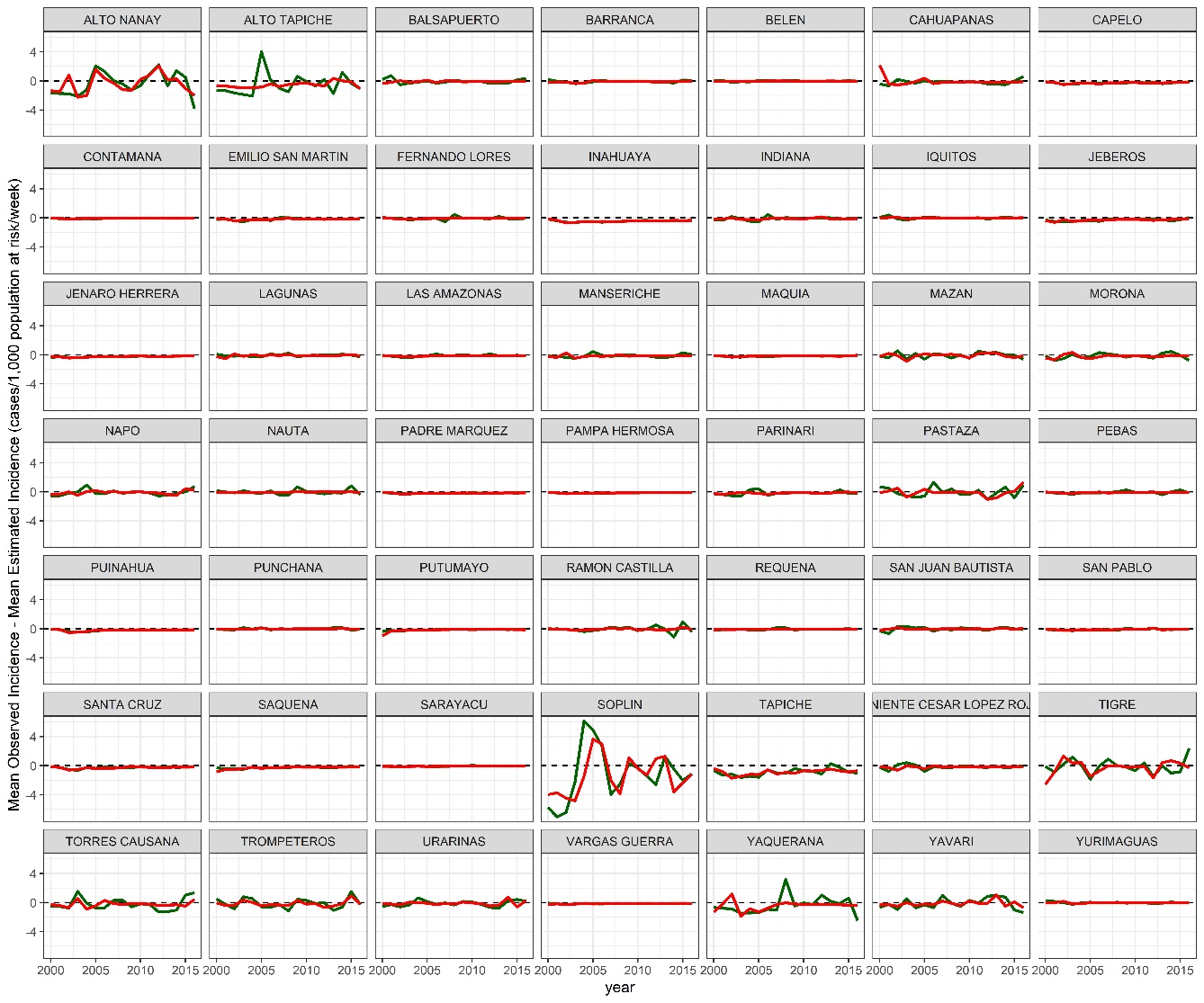 |
| Yearly average of the difference between observed incidence and modeled incidence rates (**Dark green line: P vivax; red line: P. falciparum**) |

# Supplemental Table 1

| **Supplementary Table 1: Model fit statistics (WAIC) for *P. vivax* and *P. falciparum* models** | | |
| --- | --- | --- |
|  | *P. vivax* | *P. falciparum* |
| Intervention coding scheme | WAIC | WAIC |
| Lagged and assumed to remain effective for an additional year | **395031** | **237071** |
| LLIN lagged and assumed to remain effective for an additional year; all other interventions reported as in Soto et al.^1^ | 395477 | 237452 |

# Supplemental Table 2

| Supplemental Table 2. LLIN Incidence rate ratios by district for *P. falciparum* and *P. vivax*. | | | | | | |
| --- | --- | --- | --- | --- | --- | --- |
|  | *P. falciparum* | | | *P. vivax* | | |
| District | IRR | lower | upper | IRR | lower | upper |
| ALTO NANAY | 0.98 | 0.95 | 1.01 | 0.93 | 0.90 | 0.97 |
| ALTO TAPICHE | 0.95 | 0.93 | 0.97 | 0.87 | 0.86 | 0.89 |
| BALSAPUERTO | 0.86 | 0.84 | 0.88 | 0.81 | 0.8 | 0.83 |
| BARRANCA | 1.03 | 1.00 | 1.05 | 0.94 | 0.92 | 0.95 |
| BELEN | 0.98 | 0.95 | 1.01 | 0.95 | 0.92 | 0.98 |
| CAHUAPANAS | 1.02 | 1.00 | 1.05 | 1.05 | 1.03 | 1.08 |
| CAPELO | 0.90 | 0.77 | 1.05 | 0.89 | 0.77 | 1.04 |
| CONTAMANA | 0.87 | 0.74 | 1.01 | 0.86 | 0.74 | 1.00 |
| EMILIO SAN MARTIN | 0.95 | 0.93 | 0.98 | 0.95 | 0.92 | 0.97 |
| FERNANDO LORES | 1.00 | 0.97 | 1.02 | 0.94 | 0.92 | 0.95 |
| INAHUAYA | 0.92 | 0.79 | 1.08 | 0.93 | 0.79 | 1.08 |
| INDIANA | 1.06 | 1.03 | 1.08 | 0.92 | 0.91 | 0.94 |
| IQUITOS | 1.04 | 1.00 | 1.07 | 0.99 | 0.96 | 1.02 |
| JEBEROS | 0.94 | 0.91 | 0.98 | 0.99 | 0.96 | 1.03 |
| JENARO HERRERA | 0.88 | 0.76 | 1.03 | 0.87 | 0.74 | 1.01 |
| LAGUNAS | 0.93 | 0.91 | 0.95 | 0.87 | 0.86 | 0.89 |
| LAS AMAZONAS | 0.97 | 0.95 | 0.99 | 0.95 | 0.93 | 0.96 |
| MANSERICHE | 0.97 | 0.95 | 1.00 | 0.91 | 0.89 | 0.93 |
| MAQUIA | 0.88 | 0.75 | 1.02 | 0.87 | 0.75 | 1.02 |
| MAZAN | 0.98 | 0.94 | 1.01 | 0.97 | 0.93 | 1.00 |
| MORONA | 0.97 | 0.95 | 0.99 | 0.89 | 0.87 | 0.90 |
| NAPO | 0.90 | 0.88 | 0.91 | 0.90 | 0.89 | 0.91 |
| NAUTA | 0.97 | 0.95 | 0.99 | 0.85 | 0.84 | 0.86 |
| PADRE MARQUEZ | 0.91 | 0.78 | 1.06 | 0.91 | 0.78 | 1.06 |
| PAMPA HERMOSA | 0.91 | 0.78 | 1.06 | 0.92 | 0.78 | 1.07 |
| PARINARI | 0.98 | 0.95 | 1.01 | 0.96 | 0.94 | 0.99 |
| PASTAZA | 0.87 | 0.85 | 0.88 | 0.91 | 0.90 | 0.91 |
| PEBAS | 0.99 | 0.96 | 1.03 | 1.01 | 0.99 | 1.03 |
| PUINAHUA | 0.91 | 0.78 | 1.07 | 0.92 | 0.79 | 1.07 |
| PUNCHANA | 1.01 | 0.98 | 1.05 | 0.95 | 0.92 | 0.98 |
| PUTUMAYO | 0.98 | 0.94 | 1.01 | 1.01 | 0.97 | 1.04 |
| RAMON CASTILLA | 0.90 | 0.88 | 0.92 | 0.90 | 0.89 | 0.92 |
| REQUENA | 0.99 | 0.96 | 1.02 | 0.92 | 0.89 | 0.95 |
| SAN JUAN BAUTISTA | 1.03 | 1.00 | 1.07 | 0.98 | 0.95 | 1.01 |
| SAN PABLO | 1.04 | 1.01 | 1.07 | 1.00 | 0.98 | 1.02 |
| SANTA CRUZ | 0.99 | 0.96 | 1.01 | 1.03 | 1.00 | 1.05 |
| SAQUENA | 0.91 | 0.78 | 1.06 | 0.90 | 0.77 | 1.05 |
| SARAYACU | 0.92 | 0.78 | 1.07 | 0.96 | 0.82 | 1.12 |
| SOPLIN | 1.02 | 1.00 | 1.04 | 0.97 | 0.95 | 0.98 |
| TAPICHE | 0.97 | 0.95 | 1.00 | 0.96 | 0.94 | 0.98 |
| TENIENTE CESAR LOPEZ ROJAS | 0.99 | 0.97 | 1.01 | 0.96 | 0.94 | 0.98 |
| TIGRE | 0.92 | 0.91 | 0.94 | 0.96 | 0.95 | 0.97 |
| TORRES CAUSANA | 0.97 | 0.95 | 1.00 | 0.90 | 0.88 | 0.93 |
| TROMPETEROS | 0.93 | 0.90 | 0.95 | 0.98 | 0.96 | 0.99 |
| URARINAS | 0.93 | 0.91 | 0.95 | 0.91 | 0.90 | 0.92 |
| VARGAS GUERRA | 0.93 | 0.80 | 1.08 | 0.93 | 0.80 | 1.09 |
| YAQUERANA | 1.02 | 0.98 | 1.05 | 0.98 | 0.94 | 1.01 |
| YAVARI | 0.94 | 0.92 | 0.95 | 1.02 | 1.01 | 1.03 |
| YURIMAGUAS | 1.04 | 1.02 | 1.06 | 0.92 | 0.91 | 0.94 |

# Supplemental Table 3

| Supplemental Table 3. Environmental Management Incidence rate ratios by district for *P. vivax* and *P. falciparum.* | | | | | | |
| --- | --- | --- | --- | --- | --- | --- |
|  | *P. falciparum* | | | *P. vivax* | | |
| District Name | IRR | lower | upper | IRR | lower | upper |
| ALTO NANAY | 0.98 | 0.95 | 1.01 | 0.93 | 0.90 | 0.97 |
| ALTO TAPICHE | 0.91 | 0.78 | 1.07 | 0.98 | 0.84 | 1.15 |
| BALSAPUERTO | 0.89 | 0.88 | 0.90 | 1.25 | 1.23 | 1.28 |
| BARRANCA | 0.98 | 0.95 | 1.01 | 1.04 | 1.02 | 1.06 |
| BELEN | 0.98 | 0.95 | 1.01 | 0.95 | 0.92 | 0.98 |
| CAHUAPANAS | 0.99 | 0.96 | 1.02 | 0.90 | 0.88 | 0.92 |
| CAPELO | 0.90 | 0.77 | 1.05 | 0.89 | 0.77 | 1.04 |
| CONTAMANA | 0.87 | 0.74 | 1.01 | 0.86 | 0.74 | 1.00 |
| EMILIO SAN MARTIN | 0.9 | 0.77 | 1.04 | 0.92 | 0.79 | 1.08 |
| FERNANDO LORES | 0.88 | 0.76 | 1.03 | 0.92 | 0.79 | 1.07 |
| INAHUAYA | 0.92 | 0.79 | 1.08 | 0.93 | 0.79 | 1.08 |
| INDIANA | 0.87 | 0.75 | 1.01 | 0.93 | 0.80 | 1.08 |
| IQUITOS | 1.04 | 1.00 | 1.07 | 0.99 | 0.96 | 1.02 |
| JEBEROS | 0.99 | 0.96 | 1.02 | 1.03 | 1.00 | 1.05 |
| JENARO HERRERA | 0.88 | 0.76 | 1.03 | 0.87 | 0.74 | 1.01 |
| LAGUNAS | 0.93 | 0.80 | 1.09 | 0.91 | 0.78 | 1.06 |
| LAS AMAZONAS | 0.88 | 0.76 | 1.03 | 0.95 | 0.82 | 1.11 |
| MANSERICHE | 0.99 | 0.96 | 1.01 | 1.07 | 1.05 | 1.09 |
| MAQUIA | 0.88 | 0.75 | 1.02 | 0.87 | 0.75 | 1.02 |
| MAZAN | 0.98 | 0.94 | 1.01 | 0.97 | 0.93 | 1.00 |
| MORONA | 0.95 | 0.93 | 0.97 | 0.90 | 0.89 | 0.92 |
| NAPO | 0.95 | 0.94 | 0.97 | 0.95 | 0.94 | 0.96 |
| NAUTA | 0.89 | 0.76 | 1.04 | 0.99 | 0.85 | 1.16 |
| PADRE MARQUEZ | 0.91 | 0.78 | 1.06 | 0.91 | 0.78 | 1.06 |
| PAMPA HERMOSA | 0.91 | 0.78 | 1.06 | 0.92 | 0.78 | 1.07 |
| PARINARI | 0.88 | 0.75 | 1.03 | 0.92 | 0.79 | 1.07 |
| PASTAZA | 1.00 | 0.99 | 1.02 | 1.09 | 1.08 | 1.10 |
| PEBAS | 0.88 | 0.76 | 1.03 | 1.04 | 0.89 | 1.21 |
| PUINAHUA | 0.91 | 0.78 | 1.07 | 0.92 | 0.79 | 1.07 |
| PUNCHANA | 1.01 | 0.98 | 1.05 | 0.95 | 0.92 | 0.98 |
| PUTUMAYO | 0.92 | 0.79 | 1.08 | 0.92 | 0.79 | 1.08 |
| RAMON CASTILLA | 0.94 | 0.81 | 1.10 | 1.00 | 0.86 | 1.16 |
| REQUENA | 0.88 | 0.76 | 1.03 | 0.92 | 0.79 | 1.08 |
| SAN JUAN BAUTISTA | 1.03 | 1.00 | 1.07 | 0.98 | 0.95 | 1.01 |
| SAN PABLO | 0.88 | 0.75 | 1.02 | 0.91 | 0.78 | 1.06 |
| SANTA CRUZ | 0.89 | 0.76 | 1.04 | 0.91 | 0.78 | 1.06 |
| SAQUENA | 0.91 | 0.78 | 1.06 | 0.90 | 0.77 | 1.05 |
| SARAYACU | 0.92 | 0.78 | 1.07 | 0.96 | 0.82 | 1.12 |
| SOPLIN | 1.02 | 0.87 | 1.18 | 1.06 | 0.91 | 1.24 |
| TAPICHE | 0.90 | 0.77 | 1.05 | 0.92 | 0.79 | 1.08 |
| TENIENTE CESAR LOPEZ ROJAS | 0.92 | 0.79 | 1.07 | 0.91 | 0.78 | 1.06 |
| TIGRE | 0.98 | 0.95 | 1.01 | 0.95 | 0.92 | 0.98 |
| TORRES CAUSANA | 0.94 | 0.91 | 0.97 | 0.94 | 0.92 | 0.97 |
| TROMPETEROS | 0.91 | 0.78 | 1.07 | 0.95 | 0.81 | 1.11 |
| URARINAS | 1.02 | 0.99 | 1.06 | 1.00 | 0.96 | 1.03 |
| VARGAS GUERRA | 0.93 | 0.80 | 1.08 | 0.93 | 0.80 | 1.09 |
| YAQUERANA | 0.89 | 0.76 | 1.04 | 1.06 | 0.91 | 1.24 |
| YAVARI | 0.99 | 0.96 | 1.02 | 0.95 | 0.92 | 0.98 |
| YURIMAGUAS | 1.02 | 0.99 | 1.04 | 1.07 | 1.06 | 1.09 |

# STROBE Statement—checklist of items that should be included in reports of observational studies

|  | Item No | Recommendation | Page  No |
| --- | --- | --- | --- |
| **Title and abstract** | 1 | (*a*) Indicate the study’s design with a commonly used term in the title or the abstract  **We note that this is a retrospective, observational, spatial interrupted time series analysis** | Title |
|  |  | (*b*) Provide in the abstract an informative and balanced summary of what was done and what was found  **We state that we sought to identify the impact of the PAMAFRO program in the Loreto region of Peru using hierarchical Bayesian methods** | Title |
| Introduction | | | |
| Background/rationale | 2 | Explain the scientific background and rationale for the investigation being reported  **We observe that despite considerable progress in reducing malaria transmission worldwide, the Amazon region has seen increases in malaria cases. Further, we note that these increases appear to be related to the withdrawal of funds for malaria control. The rationale for this study is that understanding malaria incidence in the context of the funding environment is critical to the future of malaria control and elimination.** | 1 |
| Objectives | 3 | State specific objectives, including any prespecified hypotheses  **Our objective is to estimate the impact of interventions supported by the PAMAFRO program in the Loreto region of Peru. Our hypothesis is that the decline in malaria transmission is due to intervention support from the program, and that the malaria resurgence in Loreto is due to the withdrawal of Global Fund support.** | 2 |
| Methods | | | |
| Study design | 4 | Present key elements of study design early in the paper  **We describe the study design and population in the first paragraph of the methods section** | 2 |
| Setting | 5 | Describe the setting, locations, and relevant dates, including periods of recruitment, exposure, follow-up, and data collection  **Sentence 1, paragraph 1 of methods section** | 2 |
| Participants | 6 | (*a*) *Cohort study*—Give the eligibility criteria, and the sources and methods of selection of participants. Describe methods of follow-up  *Case-control study*—Give the eligibility criteria, and the sources and methods of case ascertainment and control selection. Give the rationale for the choice of cases and controls  *Cross-sectional study*—Give the eligibility criteria, and the sources and methods of selection of participants |  |
|  |  | (*b*) *Cohort study*—For matched studies, give matching criteria and number of exposed and unexposed  *Case-control study*—For matched studies, give matching criteria and the number of controls per case  **NA; this is an observational study of routine surveillance data.** |  |
| Variables | 7 | Clearly define all outcomes, exposures, predictors, potential confounders, and effect modifiers. Give diagnostic criteria, if applicable  **We describe these (as relevant) in the first 3 paragraphs of the methods section** | 2 |
| Data sources/ measurement | 8* | For each variable of interest, give sources of data and details of methods of assessment (measurement). Describe comparability of assessment methods if there is more than one group  **We describe each of these in the first 3 paragraphs of the methods section** | *2* |
| Bias | 9 | Describe any efforts to address potential sources of bias  **We note that we introduce spatially varying effects to address several sources of bias that might be induced due to unobserved confounding** | 3 |
| Study size | 10 | Explain how the study size was arrived at  **This study recorded malaria case counts from routine surveillance. We further restricted our analysis to the 5 years preceding the intervention, the 5 years the intervention was administered, and the year subsequent to allow the durability of interventions to wane.** | 2 |
| Quantitative variables | 11 | Explain how quantitative variables were handled in the analyses. If applicable, describe which groupings were chosen and why  **This is done in the description of each variable** | 2 |
| Statistical methods | 12 | (*a*) Describe all statistical methods, including those used to control for confounding  **We use a hierarchical Bayesian model. Environmental variables adjust for confounding, since, for example, 1) individuals may be more likely to sleep under a bed net during the rainy season, 2) interventions are more likely to be administered during the rainy season, and 3) malaria transmission is generally higher during the end of the rainy season.** | 3 |
|  |  | (*b*) Describe any methods used to examine subgroups and interactions  **NA** |  |
|  |  | (*c*) Explain how missing data were addressed  **We did not encounter missing data** |  |
|  |  | (*d*) *Cohort study*—If applicable, explain how loss to follow-up was addressed  *Case-control study*—If applicable, explain how matching of cases and controls was addressed  *Cross-sectional study*—If applicable, describe analytical methods taking account of sampling strategy |  |
|  |  | (*e*) Describe any sensitivity analyses  **We considered different model specifications and compared model fit** |  |

| Results | | | |
| --- | --- | --- | --- |
| Participants | 13* | (a) Report numbers of individuals at each stage of study—eg numbers potentially eligible, examined for eligibility, confirmed eligible, included in the study, completing follow-up, and analysed |  |
|  |  | (b) Give reasons for non-participation at each stage |  |
|  |  | (c) Consider use of a flow diagram |  |
| Descriptive data | 14* | (a) Give characteristics of study participants (eg demographic, clinical, social) and information on exposures and potential confounders  **Unfortunately, demographic, clinical, and social information was not available from the surveillance system.** |  |
|  |  | (b) Indicate number of participants with missing data for each variable of interest  **NA** |  |
|  |  | (c) *Cohort study*—Summarise follow-up time (eg, average and total amount) |  |
| Outcome data | 15* | *Cohort study*—Report numbers of outcome events or summary measures over time |  |
|  |  | *Case-control study—*Report numbers in each exposure category, or summary measures of exposure |  |
|  |  | *Cross-sectional study—*Report numbers of outcome events or summary measures |  |
| Main results | 16 | (*a*) Give unadjusted estimates and, if applicable, confounder-adjusted estimates and their precision (eg, 95% confidence interval). Make clear which confounders were adjusted for and why they were included  **We do not provide unadjusted estimates** |  |
|  |  | (*b*) Report category boundaries when continuous variables were categorized  **NA** |  |
|  |  | (*c*) If relevant, consider translating estimates of relative risk into absolute risk for a meaningful time period  **We present annualized incidence rate ratios.** |  |
| Other analyses | 17 | Report other analyses done—eg analyses of subgroups and interactions, and sensitivity analyses  **NA** |  |
| Discussion | | | |
| Key results | 18 | Summarise key results with reference to study objectives  **We note that the PAMAFRO program had a major impact on reducing malaria transmission in Loreto, but that these results varied by district, by intervention, and by *Plasmodium*** **species.** |  |
| Limitations | 19 | Discuss limitations of the study, taking into account sources of potential bias or imprecision. Discuss both direction and magnitude of any potential bias  **We describe a number of limitations, including a lack of demographic information about the study population and its behaviors (e.g. migration), relying on passive surveillance data, etc.** |  |
| Interpretation | 20 | Give a cautious overall interpretation of results considering objectives, limitations, multiplicity of analyses, results from similar studies, and other relevant evidence  **We contextualize our findings by noting that our study is in agreement with a multi-country systematic review that indicates that 91% of malaria resurgence events are due in part to funding withdrawals.** |  |
| Generalisability | 21 | Discuss the generalisability (external validity) of the study results  **We note that our findings are likely generalizeable to the broader Amazon region since: 1) the PAMAFRO program was implemented across multiple countries in the region, and 2) the ecological conditions across the region are similar to those in Loreto.** |  |
| Other information | | | |
| Funding | 22 | Give the source of funding and the role of the funders for the present study and, if applicable, for the original study on which the present article is based  **We note our funding sources and the role of the funders in the “Role of Funding Source” section.** |  |

*Give information separately for cases and controls in case-control studies and, if applicable, for exposed and unexposed groups in cohort and cross-sectional studies.

**Note:** An Explanation and Elaboration article discusses each checklist item and gives methodological background and published examples of transparent reporting. The STROBE checklist is best used in conjunction with this article (freely available on the Web sites of PLoS Medicine at http://www.plosmedicine.org/, Annals of Internal Medicine at http://www.annals.org/, and Epidemiology at http://www.epidem.com/). Information on the STROBE Initiative is available at www.strobe-statement.org.

# Supplemental Table 4.

| Supplemental Table 4. Strengthening diagnostics incidence rate ratios by district for *P. vivax* and *P. falciparum.* | | | | | | |
| --- | --- | --- | --- | --- | --- | --- |
|  | *P. falciparum* | | | *P. vivax* | | |
| District Name | IRR | lower | upper | IRR | lower | upper |
| ALTO NANAY | 0.98 | 0.95 | 1.01 | 1.02 | 0.99 | 1.05 |
| ALTO TAPICHE | 0.94 | 0.91 | 0.97 | 0.92 | 0.90 | 0.94 |
| BALSAPUERTO | 1.04 | 1.00 | 1.07 | 0.99 | 0.95 | 1.02 |
| BARRANCA | 0.95 | 0.92 | 0.98 | 0.99 | 0.96 | 1.02 |
| BELEN | 1.01 | 0.98 | 1.05 | 1.02 | 0.99 | 1.05 |
| CAHUAPANAS | 0.93 | 0.90 | 0.97 | 0.98 | 0.95 | 1.01 |
| CAPELO | 0.90 | 0.77 | 1.05 | 0.89 | 0.77 | 1.04 |
| CONTAMANA | 0.98 | 0.97 | 1.00 | 0.98 | 0.96 | 1.00 |
| EMILIO SAN MARTIN | 0.90 | 0.77 | 1.04 | 0.92 | 0.79 | 1.08 |
| FERNANDO LORES | 0.99 | 0.97 | 1.01 | 1.14 | 1.13 | 1.16 |
| INAHUAYA | 0.92 | 0.79 | 1.08 | 0.93 | 0.79 | 1.08 |
| INDIANA | 0.97 | 0.96 | 0.99 | 0.99 | 0.97 | 1.00 |
| IQUITOS | 0.97 | 0.94 | 1.00 | 1.03 | 1.00 | 1.06 |
| JEBEROS | 0.94 | 0.92 | 0.97 | 1.02 | 0.99 | 1.04 |
| JENARO HERRERA | 0.97 | 0.95 | 0.99 | 0.98 | 0.96 | 1.00 |
| LAGUNAS | 0.95 | 0.93 | 0.97 | 1.07 | 1.05 | 1.09 |
| LAS AMAZONAS | 0.94 | 0.92 | 0.97 | 0.91 | 0.90 | 0.93 |
| MANSERICHE | 0.99 | 0.96 | 1.01 | 1.02 | 1.00 | 1.04 |
| MAQUIA | 0.99 | 0.97 | 1.02 | 1.04 | 1.02 | 1.06 |
| MAZAN | 0.98 | 0.94 | 1.01 | 0.97 | 0.93 | 1.00 |
| MORONA | 0.97 | 0.94 | 1.00 | 0.99 | 0.96 | 1.03 |
| NAPO | 1.03 | 1.01 | 1.04 | 0.99 | 0.98 | 1.00 |
| NAUTA | 0.97 | 0.94 | 1.00 | 1.02 | 0.99 | 1.06 |
| PADRE MARQUEZ | 0.91 | 0.78 | 1.06 | 0.91 | 0.78 | 1.06 |
| PAMPA HERMOSA | 0.91 | 0.78 | 1.06 | 0.92 | 0.78 | 1.07 |
| PARINARI | 0.93 | 0.91 | 0.95 | 0.91 | 0.89 | 0.92 |
| PASTAZA | 0.93 | 0.90 | 0.96 | 0.93 | 0.91 | 0.96 |
| PEBAS | 0.99 | 0.95 | 1.04 | 0.97 | 0.93 | 1.01 |
| PUINAHUA | 1.00 | 0.98 | 1.02 | 1.02 | 1.00 | 1.04 |
| PUNCHANA | 0.93 | 0.91 | 0.95 | 1.09 | 1.07 | 1.10 |
| PUTUMAYO | 0.98 | 0.96 | 1.01 | 0.99 | 0.96 | 1.02 |
| RAMON CASTILLA | 1.01 | 0.97 | 1.04 | 1.02 | 0.99 | 1.06 |
| REQUENA | 0.99 | 0.96 | 1.02 | 0.99 | 0.96 | 1.03 |
| SAN JUAN BAUTISTA | 0.96 | 0.93 | 0.99 | 0.95 | 0.92 | 0.97 |
| SAN PABLO | 0.98 | 0.97 | 1.00 | 1.05 | 1.03 | 1.07 |
| SANTA CRUZ | 0.89 | 0.76 | 1.04 | 0.91 | 0.78 | 1.06 |
| SAQUENA | 0.98 | 0.96 | 1.00 | 0.97 | 0.95 | 0.99 |
| SARAYACU | 0.99 | 0.96 | 1.01 | 0.93 | 0.91 | 0.95 |
| SOPLIN | 0.96 | 0.93 | 0.98 | 0.92 | 0.90 | 0.94 |
| TAPICHE | 0.90 | 0.77 | 1.05 | 0.92 | 0.79 | 1.08 |
| TENIENTE CESAR LOPEZ ROJAS | 0.95 | 0.93 | 0.96 | 0.91 | 0.90 | 0.93 |
| TIGRE | 0.97 | 0.95 | 0.98 | 0.96 | 0.95 | 0.97 |
| TORRES CAUSANA | 0.98 | 0.94 | 1.02 | 1.00 | 0.96 | 1.04 |
| TROMPETEROS | 1.03 | 1.01 | 1.04 | 1.07 | 1.06 | 1.08 |
| URARINAS | 0.94 | 0.92 | 0.96 | 0.96 | 0.94 | 0.97 |
| VARGAS GUERRA | 0.99 | 0.96 | 1.01 | 0.97 | 0.95 | 1.00 |
| YAQUERANA | 1.06 | 1.02 | 1.10 | 1.01 | 0.98 | 1.05 |
| YAVARI | 0.99 | 0.96 | 1.02 | 0.95 | 0.92 | 0.98 |
| YURIMAGUAS | 0.92 | 0.90 | 0.95 | 0.95 | 0.92 | 0.98 |

# Supplemental Table 5.

| Supplementary Table 5. Training health worker incidence rate ratios by district for *P. falciparum* and *P. vivax.* | | | | | | |
| --- | --- | --- | --- | --- | --- | --- |
|  | *P. falciparum* | | | *P. vivax* | | |
| District Name | IRR | lower | upper | IRR | lower | upper |
| ALTO NANAY | 0.98 | 0.95 | 1.01 | 1.02 | 0.99 | 1.05 |
| ALTO TAPICHE | 1.03 | 1.00 | 1.06 | 1.09 | 1.07 | 1.11 |
| BALSAPUERTO | 1.04 | 1.00 | 1.07 | 0.99 | 0.95 | 1.02 |
| BARRANCA | 0.95 | 0.92 | 0.98 | 0.99 | 0.96 | 1.02 |
| BELEN | 1.01 | 0.98 | 1.05 | 1.02 | 0.99 | 1.05 |
| CAHUAPANAS | 0.93 | 0.90 | 0.97 | 0.98 | 0.95 | 1.01 |
| CAPELO | 0.90 | 0.77 | 1.05 | 0.89 | 0.77 | 1.04 |
| CONTAMANA | 0.87 | 0.74 | 1.01 | 0.86 | 0.74 | 1.00 |
| EMILIO SAN MARTIN | 0.98 | 0.96 | 1.00 | 1.02 | 1.01 | 1.04 |
| FERNANDO LORES | 0.99 | 0.96 | 1.01 | 0.90 | 0.89 | 0.91 |
| INAHUAYA | 0.92 | 0.79 | 1.08 | 0.93 | 0.79 | 1.08 |
| INDIANA | 0.98 | 0.96 | 1.01 | 1.02 | 1.00 | 1.03 |
| IQUITOS | 0.97 | 0.94 | 1.00 | 1.03 | 1.00 | 1.06 |
| JEBEROS | 0.98 | 0.96 | 1.01 | 0.99 | 0.97 | 1.02 |
| JENARO HERRERA | 0.88 | 0.76 | 1.03 | 0.87 | 0.74 | 1.01 |
| LAGUNAS | 0.99 | 0.97 | 1.01 | 0.98 | 0.97 | 1.00 |
| LAS AMAZONAS | 0.88 | 0.76 | 1.03 | 0.95 | 0.82 | 1.11 |
| MANSERICHE | 0.98 | 0.95 | 1.01 | 0.92 | 0.90 | 0.95 |
| MAQUIA | 0.88 | 0.75 | 1.02 | 0.87 | 0.75 | 1.02 |
| MAZAN | 1.06 | 1.05 | 1.07 | 1.05 | 1.04 | 1.06 |
| MORONA | 0.97 | 0.94 | 1.00 | 0.99 | 0.96 | 1.03 |
| NAPO | 0.98 | 0.96 | 0.99 | 0.97 | 0.97 | 0.98 |
| NAUTA | 0.97 | 0.94 | 1.00 | 1.02 | 0.99 | 1.06 |
| PADRE MARQUEZ | 0.91 | 0.78 | 1.06 | 0.91 | 0.78 | 1.06 |
| PAMPA HERMOSA | 0.91 | 0.78 | 1.06 | 0.92 | 0.78 | 1.07 |
| PARINARI | 1.00 | 0.97 | 1.03 | 1.00 | 0.98 | 1.03 |
| PASTAZA | 0.93 | 0.90 | 0.96 | 0.93 | 0.91 | 0.96 |
| PEBAS | 0.99 | 0.95 | 1.04 | 0.97 | 0.93 | 1.01 |
| PUINAHUA | 0.91 | 0.78 | 1.07 | 0.92 | 0.79 | 1.07 |
| PUNCHANA | 0.96 | 0.95 | 0.98 | 0.88 | 0.88 | 0.89 |
| PUTUMAYO | 0.98 | 0.95 | 1.01 | 0.98 | 0.95 | 1.00 |
| RAMON CASTILLA | 1.01 | 0.97 | 1.04 | 1.02 | 0.99 | 1.06 |
| REQUENA | 0.97 | 0.94 | 1.01 | 1.10 | 1.06 | 1.13 |
| SAN JUAN BAUTISTA | 0.96 | 0.93 | 0.99 | 0.95 | 0.92 | 0.97 |
| SAN PABLO | 0.88 | 0.75 | 1.02 | 0.91 | 0.78 | 1.06 |
| SANTA CRUZ | 0.89 | 0.76 | 1.04 | 0.91 | 0.78 | 1.06 |
| SAQUENA | 0.91 | 0.78 | 1.06 | 0.90 | 0.77 | 1.05 |
| SARAYACU | 0.92 | 0.78 | 1.07 | 0.96 | 0.82 | 1.12 |
| SOPLIN | 1.02 | 1.00 | 1.04 | 1.03 | 1.01 | 1.05 |
| TAPICHE | 0.90 | 0.77 | 1.05 | 0.92 | 0.79 | 1.08 |
| TENIENTE CESAR LOPEZ ROJAS | 0.92 | 0.79 | 1.07 | 0.91 | 0.78 | 1.06 |
| TIGRE | 0.98 | 0.95 | 1.01 | 0.95 | 0.92 | 0.98 |
| TORRES CAUSANA | 0.98 | 0.94 | 1.02 | 1.00 | 0.96 | 1.04 |
| TROMPETEROS | 0.91 | 0.78 | 1.07 | 0.95 | 0.81 | 1.11 |
| URARINAS | 1.02 | 0.99 | 1.06 | 1.00 | 0.96 | 1.03 |
| VARGAS GUERRA | 0.93 | 0.80 | 1.08 | 0.93 | 0.80 | 1.09 |
| YAQUERANA | 1.06 | 1.02 | 1.10 | 1.05 | 1.01 | 1.08 |
| YAVARI | 1.04 | 1.02 | 1.06 | 1.04 | 1.03 | 1.05 |
| YURIMAGUAS | 0.92 | 0.90 | 0.95 | 0.95 | 0.92 | 0.98 |
